# RNA Polymerase II Stalling Promotes Nucleosome Occlusion and pTEFb Recruitment to Drive Immortalization by Epstein-Barr Virus

**DOI:** 10.1371/journal.ppat.1002334

**Published:** 2011-10-27

**Authors:** Richard D. Palermo, Helen M. Webb, Michelle J. West

**Affiliations:** School of Life Sciences, University of Sussex, Falmer, Brighton, United Kingdom; Wistar Institute, United States of America

## Abstract

Epstein-Barr virus (EBV) immortalizes resting B-cells and is a key etiologic agent in the development of numerous cancers. The essential EBV-encoded protein EBNA 2 activates the viral C promoter (Cp) producing a message of ∼120 kb that is differentially spliced to encode all EBNAs required for immortalization. We have previously shown that EBNA 2-activated transcription is dependent on the activity of the RNA polymerase II (pol II) C-terminal domain (CTD) kinase pTEFb (CDK9/cyclin T1). We now demonstrate that Cp, in contrast to two shorter EBNA 2-activated viral genes (LMP 1 and 2A), displays high levels of promoter-proximally stalled pol II despite being constitutively active. Consistent with pol II stalling, we detect considerable pausing complex (NELF/DSIF) association with Cp. Significantly, we observe substantial Cp-specific pTEFb recruitment that stimulates high-level pol II CTD serine 2 phosphorylation at distal regions (up to +75 kb), promoting elongation. We reveal that Cp-specific pol II accumulation is directed by DNA sequences unfavourable for nucleosome assembly that increase TBP access and pol II recruitment. Stalled pol II then maintains Cp nucleosome depletion. Our data indicate that pTEFb is recruited to Cp by the bromodomain protein Brd4, with polymerase stalling facilitating stable association of pTEFb. The Brd4 inhibitor JQ1 and the pTEFb inhibitors DRB and Flavopiridol significantly reduce Cp, but not LMP1 transcript production indicating that Brd4 and pTEFb are required for Cp transcription. Taken together our data indicate that pol II stalling at Cp promotes transcription of essential immortalizing genes during EBV infection by (i) preventing promoter-proximal nucleosome assembly and ii) necessitating the recruitment of pTEFb thereby maintaining serine 2 CTD phosphorylation at distal regions.

## Author Summary

Epstein-Barr virus (EBV) is associated with the development of a number of human cancers including Burkitt's lymphoma, Hodgkin's lymphoma, nasopharyngeal carcinoma and post-transplant lymphoproliferative disease. The virus infects B cells rendering them immortal through the production of a small number of viral proteins in the latently infected cell. Many of the viral proteins required for B-cell immortalization are produced from a very long protein-coding RNA message that initiates at the main viral latency promoter C, and our results provide important new information on how this message is produced. Specifically we show that the production of this long RNA is driven by the recruitment of the elongation factor (pTEFb) to paused transcription complexes at the C promoter. We show that pTEFb is recruited by the chromatin-associated protein, Brd4. Treatment of cells with a recently developed Brd4 inhibitor and inhibitors of the pTEFb elongation factor inhibits production of transcripts derived from the long EBV message thereby highlighting Brd4 and pTEFb inhibitors as potential anti-EBV agents.

## Introduction

Epstein-Barr virus (EBV) is causally associated with the development of numerous tumours including Burkitt's lymphoma, Hodgkin's lymphoma, nasopharyngeal carcinoma and post-transplant lymphoproliferative disease and immortalizes resting B cells *in vitro* generating latently infected lymphoblastoid cell-lines (LCLs) [1]. LCLs express 9 viral latent proteins: EBV Nuclear Antigens (EBNAs 1, 2, 3A, 3B, 3C and LP) and three membrane proteins (LMP 1, 2A and 2B). Following initial infection, EBNA-LP and EBNA 2 are expressed from the viral W promoter (Wp). EBNA 2 then drives promoter switching through activation of the upstream viral C promoter (Cp) to produce a long message (up to 120 kb) that is differentially spliced to produce transcripts encoding all nuclear antigens required for immortalization [2]. EBNA 2 also activates two promoters that direct transcription of the EBV oncogene latent membrane protein 1 (LMP1) and the viral LMP 2A and 2B genes [3]–[4]. EBNA 2 is directed to promoters via association with the cellular DNA binding proteins RBP-Jκ and PU.1 [5]–[8]. Transcriptional activation by EBNA 2 involves the promotion of transcription initiation through associations with histone acetyltransferases [9], chromatin remodelling complexes [10]–[11], and the basal transcriptional machinery [12]–[14] and leads to Histone H3 and H4 acetylation at target gene promoters *in vivo* [15]. The association of EBNA 2 with target promoters is increased by asymmetric arginine dimethylation in the arginine-glycine repeat region of the protein [16] and is inhibited by phosphorylation on serine 243 during mitosis and viral lytic cycle [17]–[19].

The carboxy-terminal domain (CTD) of RNA Polymerase II (pol II) plays a central role in regulating efficient transcription initiation, elongation and RNA processing. It contains 52 heptapeptide repeats (Y_1_S_2_P_3_T_4_S_5_P_6_S_7_) and is phosphorylated largely on serines 2 and 5 during transcription [20]. Following pol II recruitment, promoter-proximal serine 5 CTD phosphorylation is mediated mainly by the TFIIH kinase, CDK7. Serine 2 CTD phosphorylation catalysed by CDK9/Cyclin T1 (positive transcription elongation factor b; p-TEFb) subsequently peaks at the 3′ end of genes. Using the specific inhibitors 5,6-dichloro-1-β-D-ribofuranosylbenzimidazole (DRB) and Flavopiridol, pTEFb has been shown to be required for productive elongation [21]–[22] by functioning as a CTD kinase and a regulator of the pol II-associated complexes DRB sensitivity-inducing factor (DSIF) and Negative Elongation Factor (NELF). DSIF and NELF induce promoter-proximal pausing that is relieved following the phosphorylation of DSIF, NELF and the pol II CTD by pTEFb [23]–[26]. Although NELF is localized to promoters and promoter-proximal regions *in vivo*, DSIF, in its phosphorylated form, continues to associate with pol II throughout genes and functions as a positive elongation factor capable of reducing polymerase stalling at pause sites, preventing transcript release and stimulating elongation [25], [27]. Interestingly, although initial studies in *Drosophila* documented the negative effects of NELF-induced promoter-proximal pausing on transcription of a subset of genes, including the Hsp70 locus [28]–[29], recent studies have demonstrated that the presence of NELF is required for the efficient transcription of the majority of *Drosophila* genes, forming a barrier to nucleosome assembly around the promoter [30].

Although pTEFb activity may be required for the efficient transcription of many cellular genes [22], not all gene transcription is pTEFb-dependent [31]–[32] and it is clear that many cellular and viral transactivators recruit and/or activate pTEFb to facilitate high-level gene-specific transcription elongation [33]. The bromodomain protein Brd4 can also recruit pTEFb to promoters via acetylated histones [34]–[35].

We have shown that EBNA 2 transcriptional activation requires pTEFb activity and promotes serine 5 CTD phosphorylation [36]. In this study we investigated how long-range EBV transcription required for viral immortalization is driven from the EBNA 2-responsive C promoter. Our results provide the first demonstration that significant levels of pol II accumulate specifically at Cp in association with the pausing factors DSIF and NELF and that pTEFb is recruited to the promoter at high level. Our data indicate that promoter-proximal pol II accumulation at Cp is directed by specific DNA sequences and maintains nucleosome depletion. Pausing facilitates pTEFb recruitment via Brd4 to drive high-level serine 2 CTD phosphorylation to promote production of the EBNA-encoding transcripts required for EBV immortalization.

## Results

### EBNA 2 Stimulates CTD Phosphorylation on Serine 2 and Serine 5 at Distal Genome Regions

**Figure 1:**
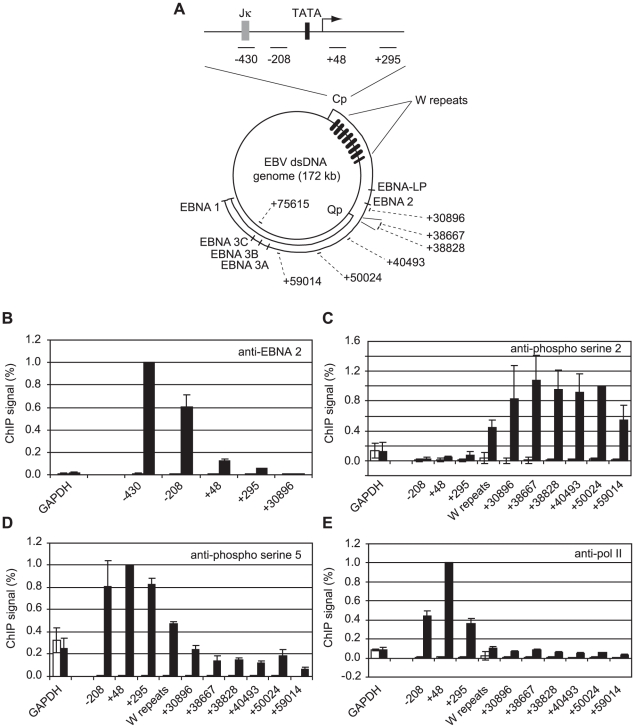
High-level pol II accumulation at Cp and CTD phosphorylation at distal EBV genome regions. (A) Diagram showing the locations of the amplicons generated by the indicated primer sets (Table S1) at the C promoter and around the circular EBV episome. Numbers indicate the 5′ end of the forward primer relative to the Cp transcription start site in the annotated B95-8 EBV sequence (NC_007605.1). The RBP-Jκ site (grey box) and TATA box (black box) are shown. (B) ChIP using anti-EBNA 2 antibodies. Percentage input signals, after subtraction of no antibody controls, are expressed relative to the highest signal obtained in all or the majority of experiments. Results show the mean +/− standard deviation of a minimum of three independent experiments carried out using at least 2 chromatin batches from Mutu I cells (open bars) and Mutu III cells (black bars). ChIP using anti-phospho serine 2 pol II CTD antibodies (C), anti-phospho serine 5 pol II CTD antibodies (D) and anti-pol II antibodies (E).

We have previously demonstrated that EBNA 2 increases serine 5 CTD phosphorylation at viral latency promoter (Cp)-proximal and downstream regions and requires pTEFb for activation of both Cp and LMP1p [36]. To examine the role of EBNA 2 in facilitating transcription of the very long primary transcript (∼120 kb) encoding all EBV nuclear antigens (EBNAs) from Cp, we probed EBNA 2-driven changes in CTD phosphorylation at distal genome regions. We examined Cp transcription in a pair of EBV-positive Burkitt's lymphoma (BL) clonal cell-lines that either maintain the original EBNA 1-only (Latency I) BL tumour phenotype (Mutu I) or have drifted in culture to express the full panel of latent antigens including EBNA 2 (Mutu III) [37]. We found that EBNA 2 binding to Cp peaked around the RBP-Jκ site in Mutu III cells and was undetectable in Mutu I cells as expected (Figure 1B). EBNA 2-activated transcription in Mutu III cells resulted in increased serine 2 CTD phosphorylation which was evident from +295 and started to increase significantly in the W repeat region of the genome (typically 7.6 repeats located +666 to +24020 downstream from Cp), remaining high up to approximately 60 kb downstream (Figure 1C). In line with our previous observations in cells expressing conditionally active EBNA 2 [36], we found that EBNA 2-activated Cp transcription resulted in large increases in pol II recruitment and serine 5 CTD phosphorylation at promoter-proximal regions consistent with the promotion of transcription initiation (Figure 1D and E). In this study we found that increased serine 5 CTD phosphorylation was maintained at distal regions (Figure 1D). ChIP assays using an antibody that precipitates total pol II detected increases in the association of pol II with distal regions in Mutu III cells compared to Mutu I cells (Figure 1E), consistent with the promotion of transcriptional elongation by EBNA 2 to drive synthesis of the full panel of EBNAs expressed in Mutu III cells. Importantly, the observed changes in distal pol II CTD phosphorylation could not be accounted for by increased pol II presence alone, since increases in phospho-epitope levels exceeded the increases in total pol II (Figure 1E). We confirmed that distal serine 2 CTD phosphorylation required functional EBNA 2 using cells expressing a conditionally-active estrogen receptor-EBNA 2 fusion protein [38]; high level serine 2 CTD phosphorylation was detectable up to 75 kb downstream from Cp only in the presence of beta-estradiol (Figure S1).

Interestingly, our experiments revealed a large peak of pol II accumulation at Cp consistent with significant pol II stalling despite the fact that Cp was constitutively active (Figure 1E). In contrast, high levels of pol II were not detectable at the alternative promoter Q (Qp, +38800 downstream from Cp) that drives EBNA 1 transcription in Mutu I cells (Figure 1A & E), despite the fact that Q was fully active (Figure S2), indicating a lack of high-level pol II recruitment or stalling at Qp.

### pTEFb Is Recruited to Cp and Is Required for Distal Pol II CTD Phosphorylation

**Figure 2:**
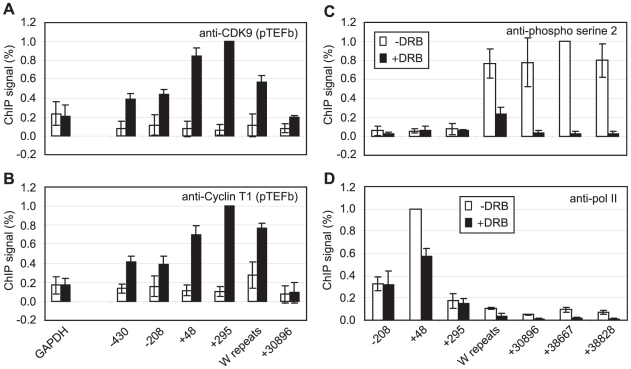
pTEFb is recruited to Cp and is required for serine 2 phosphorylation. Results show the mean +/− standard deviation of a minimum of three independent experiments carried out using at least 2 chromatin batches. Mutu I signals (open bars) are compared to Mutu III signals (black bars) in ChIP using anti-CDK9 antibodies (A) or anti-cyclin T1 antibodies (B). (C) ChIP using anti-phospho serine 2 pol II CTD antibodies in Mutu III cells minus (open bars) or plus 500 µM DRB (black bars). (D) ChIP using anti-pol II antibodies in Mutu III cells −/+ DRB.

We have previously demonstrated that EBNA 2-activated transcription requires pTEFb activity [36]. Since CDK9 predominantly phosphorylates the pol II CTD on serine 2 during elongation through association with the travelling pol II complex [39], we examined pTEFb recruitment at Cp. ChIP assays using anti-CDK9 and anti-cyclin T1 antibodies demonstrated that high levels of both subunits of pTEFb were associated with Cp in Mutu III cells (Figure 2). Consistent with a role for pTEFb in distal serine 2 CTD phosphorylation, pTEFb was detectable in the W repeats and at 31 kb downstream (Figure 2) but fell to levels below the limits of detection of our ChIP assays thereafter. Previous studies have shown that pTEFb levels can drop significantly and be barely detectable using standard ChIP methods even 2 kb downstream from promoters, despite clear evidence of pTEFb function (i.e. Serine 2 phosphorylation) at these regions [40]–[41]. To further confirm that pTEFb was the kinase responsible for pol II CTD phosphorylation beyond 31 kb, Mutu III cells were treated with the pTEFb inhibitor, DRB. Our results demonstrated that DRB ablated serine 2 phosphorylation on the pol II CTD and severely reduced polymerase retention at distal regions (Figure 2). We also observed a reduction in pol II phosphorylation on serine 5 at distal regions, supporting previous observations of a role for pTEFb in catalysing serine 5 phosphorylation during elongation (Figure S3) [39]. ChIP for Tata box binding protein (TBP) confirmed that DRB treatment did not have general non-specific effects on Cp pre-initiation complex assembly (Figure S3). DRB treatment of cells expressing conditionally active EBNA 2 also confirmed the requirement for pTEFb for distal serine 2 CTD phosphorylation and pol II retention up to 75 kb downstream during EBNA2-dependent transcription (Figure S3).

### High-levels of the Pausing Complexes NELF and DSIF Are Present at Cp

**Figure 3:**
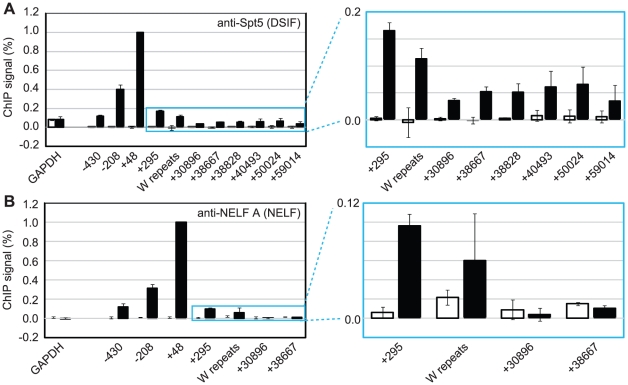
DSIF and NELF are recruited to Cp in Mutu III cells. Results show the mean +/− standard deviation of a minimum of three independent experiments carried out using at least 2 chromatin batches. Mutu I signals (open bars) are compared to Mutu III signals (black bars). ChIP using anti-Spt5 antibodies to detect DSIF (A) or anti-NELF-A antibodies to detect NELF (B). Blue-edged graphs show zoomed-in sections to allow better visualization of downstream primer signals.

To further investigate pol II stalling at Cp, we examined the association of the pausing complexes NELF and DSIF with the promoter. We detected high-levels of the NELF-A subunit of the NELF complex and the Spt5 subunit of the DSIF Spt4-Spt5 heterodimer at Cp in Mutu III cells (Figure 3) consistent with DSIF and NELF-induced polymerase stalling. Unlike NELF, which was absent from distal regions of the template, Spt5 remained detectable at distal regions consistent with a role for DSIF in promoting transcriptional elongation [25], [27] (Figure 3). EBNA 2-dependent pausing complex recruitment to Cp was confirmed in cells expressing conditionally active EBNA 2 (Figure S4). Our results suggest that recruitment of pTEFb to Cp is likely to be required to overcome stalling induced by DSIF and NELF and promote elongation to distal regions through serine 2 phosphorylation of the pol II CTD.

### Low Level Pol II CTD Serine 2 Phosphorylation and pTEFb, NELF and DSIF Recruitment at the EBNA 2-regulated LMP Gene Locus

**Figure 4:**
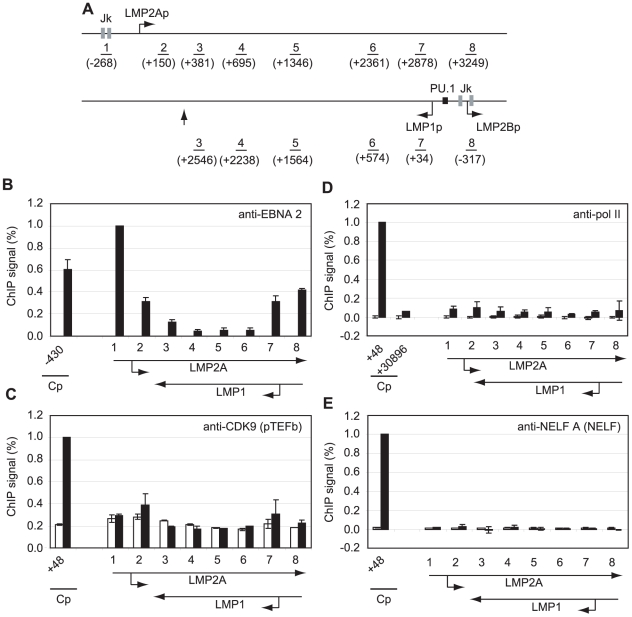
Pol II is not paused at the LMP gene loci and little pTEFb is recruited. (A) Diagram of the LMP gene locus showing the locations of the amplicons generated by the indicated primer sets (1–8). Numbers indicate the 5′ end of the forward primer relative to the starts of the LMP1 or LMP2A mRNA sequences (bent arrows) in the B95-8 annotated EBV sequence (NC_007605). The RBP-Jκ sites (grey boxes) and PU.1 site (black box) are shown. The vertical arrow indicates the position of the LMP1 polyadenylation site. Results show the mean +/− standard deviation of a minimum of two independent experiments using Mutu I (open bars) and Mutu III cell chromatin (black bars). Percentage input signals, after subtraction of no antibody controls, are expressed for comparison purposes relative to the highest signal obtained using Cp-specific primers. ChIP using anti-EBNA 2 antibodies (B), anti-CDK9 antibodies (C), anti-pol II antibodies (D) and anti-NELF-A antibodies (E).

To determine whether polymerase stalling, high level pTEFb recruitment and large increases in serine 2 CTD phosphorylation were evident at other shorter EBNA 2-responsive transcription units, we performed ChIP assays using primers specific for the EBNA 2-activated LMP genes (Figure 4). Transcription of the LMP2A gene is regulated by EBNA 2-via two RBP-Jκ sites; EBNA 2-dependent LMP1 transcription is driven by a bidirectional promoter located in the reverse orientation in the EBV genome via EBNA 2 binding to both RBP-Jκ and PU.1 (Figure 4A). This bidirectional promoter also drives transcription of the LMP2B gene. The LMP2A and LMP1 transcription units therefore overlap and ChIP assays with primer sets 3–8 detect transcription complexes associated with either or both genes (Figure 4A). ChIP assays detected the same or higher levels of EBNA 2 binding to the LMP1 and LMP2A promoters in Mutu III cells to those detected at Cp (Figure 4B). Interestingly however, pTEFb recruitment to LMP promoters was barely detectable and no pol II stalling was evident at either LMP promoter (Figure 4). To rule out the possibility that we had failed to detect a pol II peak at LMP2Ap due to the location of our primer sets (−268 to −185 and +150 to +231), we designed an additional primer set spanning the transcription start site (−50 to +34). This primer set did not detect any higher pol II signal than the flanking primer sets (Figure S5). NELF and DSIF recruitment to the LMP locus was also minimal (Figures 4 and S6). Consistently, pol II CTD serine 2 phosphorylation did not reach the high levels observed at distal Cp regions and serine 5 phosphorylation on the pol II CTD was also much reduced (Figure S6). Similar results were obtained when Cp and the LMP locus were compared in an EBV-infected LCL (Figure S7).

To exclude the possibility that low-level pol II and transcription factor association with the LMP gene locus simply reflected low-levels of LMP transcription in the cell-lines under study, we used real-time PCR to determine the levels of Cp-initiated EBNA 2 and EBNA 1 transcripts and compared these to levels of LMP1 transcripts in Mutu III cells and two EBV-infected LCLs (Figure S8). We found that LMP1 transcript levels were equivalent to the levels of Cp-initiated EBNA 2 and EBNA 1 transcripts produced in the same cell-line, although there were variations in the level of transcripts produced between cell-lines probably as a result of differences in EBV genome copy number (Figure S8).

Taken together, our data indicate that pol II accumulation and high-level pTEFb recruitment is not a general characteristic of EBNA2- activated promoters, but is specific to Cp. Moreover, the level of promoter-associated pol II does not simply reflect the level of gene transcription from Cp and LMP1p.

### Pol II Stalling at Cp Maintains a Nucleosome-depleted Region

**Figure 5:**
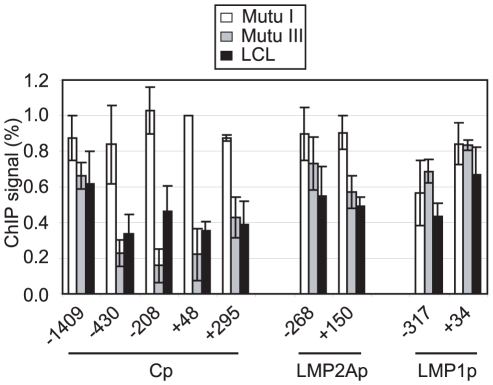
Pol II pausing at Cp prevents nucleosome assembly around the promoter. ChIP using anti-histone H3 antibodies. Results show the mean +/− standard deviation of a minimum of three independent experiments carried out using at least 2 chromatin batches in Mutu I cells (open bars), Mutu III cells (grey bars) and the PER 253 B95-8 LCL (black bars). Primer sets probed promoter-proximal regions of Cp, LMP2Ap and LMP1p. Percentage input signals, after subtraction of no antibody controls are expressed relative to the highest signal obtained in all or the majority of experiments.

Pol II stalling has recently been implicated in the promotion of gene activity through the maintenance of a promoter-proximal nucleosome-free region [30]. We therefore investigated whether the region around Cp was depleted of nucleosomes in the presence of stalled polymerase in Mutu III cells and an LCL where Cp is active, compared to Mutu I cells where Cp is inactive. Nucleosome levels were measured in ChIP assays using antibodies against the core histone, histone H3 [41]. Strikingly, we detected an 84% decrease in nucleosome occupancy at Cp in Mutu III cells compared to Mutu I cells using primer sets that spanned the region −208 to −96 bp upstream of the transcription start site and a 78% and 73% decrease with primer sets spanning regions +48 to +167 and −430 to −337, respectively (Figure 5). Nucleosomes were similarly depleted from these regions in an EBV-infected LCL (Figure 5). In contrast, levels of nucleosome depletion at similar regions around LMP2Ap and LMP1p were much lower, consistent with the absence of stalled pol II at these promoters (Figure 5). It is therefore clear that in the absence of Cp activity in Mutu I cells, nucleosomes assemble over promoter regions, but in the presence of stalled polymerase in Mutu III cells, Cp is maintained in a nucleosome-depleted state. In contrast, the low levels of pol II initiating at the LMP promoters are unable to maintain a highly nucleosome-depleted region and transient remodelling is likely to facilitate initiation.

### Pol II Stalling at Cp May Be Directed by Specific Sequences that Allow Increased Access to the Transcription Machinery

**Figure 6:**
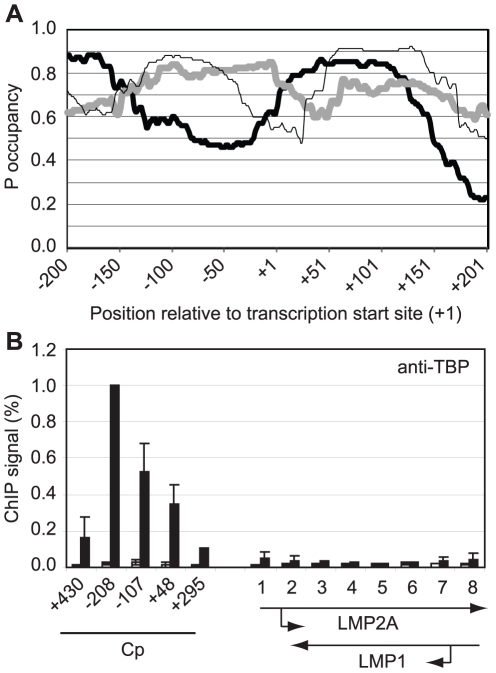
Pol II pausing at Cp is driven by DNA sequences that promote access by TBP. (A) The probability of nucleosome occupancy (P occupancy) at regions upstream and downstream from the transcription start site (TSS) of Cp (thick black line), LMP1p (thick grey line) and LMP 2Ap (thin black line) predicted using tools available at http://genie.weizmann.ac.il/software/nucleo_prediction.html [44]. (B) ChIP using anti-TBP antibodies analysed using Cp and LMP gene primers (see Figure 4) in Mutu I cells (open bars) and Mutu III cells (black bars). Results show the mean +/− standard deviation of a minimum of three independent experiments carried out using at least 2 chromatin batches. Percentage input signals, after subtraction of no antibody controls, are expressed for comparison purposes relative to the highest signal obtained at Cp.

Gene-specificity of polymerase stalling may be directed by the ability of promoters to recruit high levels of the general transcription factor TFIID and thus high levels of polymerase molecules [42]–[43]. Promoters that contain DNA sequences less favourable for nucleosome assembly may therefore be predicted to recruit TFIID and transcription complexes more efficiently and accumulate stalled pol II in association with DSIF and NELF. To test whether this could explain the specificity of polymerase stalling at Cp, we examined the propensity of the DNA sequences around Cp to assemble into nucleosomes using a nucleosome occupancy prediction program http://genie.weizmann.ac.il/software/nucleo_prediction.html [44]. This revealed a dramatic difference in the probability of nucleosome occupancy at Cp compared to the LMP promoters (Figure 6). The region of Cp encompassing the TATA signal appears much less likely to be occupied by nucleosomes compared to the equivalent regions of LMP1p and LMP2Ap (TATA boxes are located at −31 to −26, −32 to −27 and −28 to −23 at the C, LMP1 and LMP2A promoters respectively). Consistent with these predictions, ChIP assays using an anti-TBP antibody detected dramatically lower levels of TBP binding at the LMP promoters compared to Cp (Figure 6). We detected high-level TBP association around the Cp TATA box (−107 to −2) and upstream (−208 to −96) presumably as a result of cross-linked interactions between TBP (TFIID) and the transcription complex following initial TBP binding to the more accessible TATA signal. Our data are therefore in agreement with a model in which initial recruitment of high levels of pol II to Cp, presumably in association with the pol II binding factors NELF and DSIF, is driven by increased accessibility of the promoter to TBP. It is clear however, that in the absence of active Cp transcription in Mutu I cells, nucleosomes are able to assemble at Cp (Figure 5) and that the reduced probability of nucleosome occupancy may provide an initial advantage to pre-initiation complex assembly, but does not completely preclude nucleosome assembly. Moreover, the presence of stalled polymerase maintains nucleosome depletion further upstream and downstream from the Cp regions predicted to be less likely to be occupied by nucleosomes since primer sets spanning −430 to −337 and +295 to +406 detect reduced histone H3 levels (Figure 5).

### Polymerase Stalling Stabilizes pTEFb Recruitment to Cp Via Brd4

**Figure 7:**
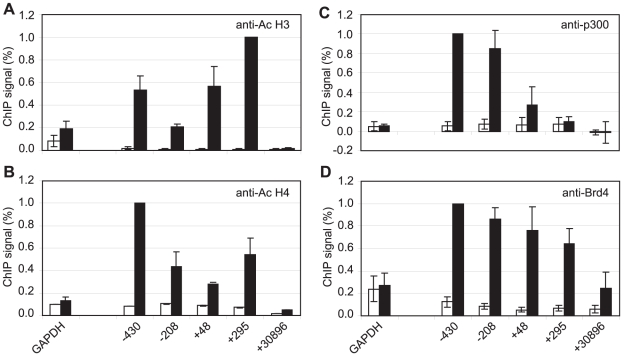
Brd4 is recruited to Cp through acetylated histones. Results show the mean +/− standard deviation of a minimum of three independent experiments carried out using at least two chromatin batches from Mutu I cells (open bars) and Mutu III cells (black bars). ChIP using anti-acetyl Histone H3 antibodies (A), anti-acetyl Histone H4 antibodies (B), anti-p300 antibodies (C) and anti-Brd4 antibodies (D).

**Figure 8:**
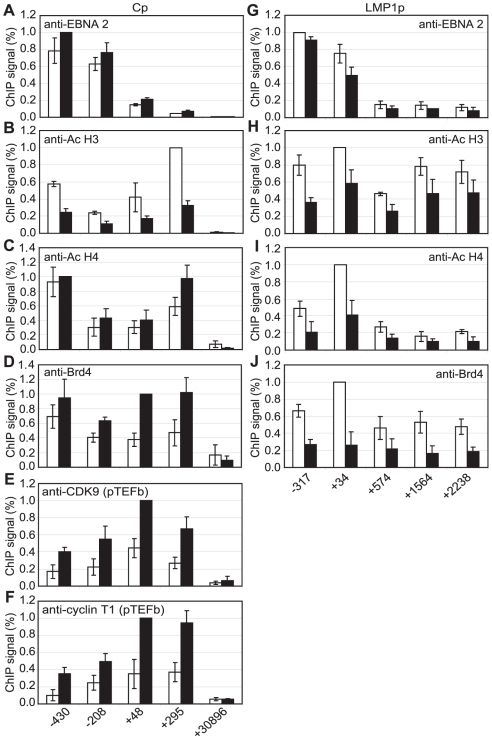
Brd4 recruits pTEFb to Cp. Results show the mean +/− standard deviation of a minimum of three independent experiments carried out using at least 2 chromatin batches from Mutu III cells in the absence (open bars) or presence of DRB (black bars). ChIP using anti-EBNA 2 (A and G), anti-acetyl Histone H3 (B and H), anti-acetyl Histone H4 (C and I), anti-Brd 4 (D and J), anti-CDK9 (E) and anti-cyclin T1 antibodies (F). Cp analysis is shown in A–F and LMP1p/LMP 2A analysis in shown in G–J.

Although pTEFb can be recruited to promoters via association with activators, we have been unable to demonstrate binding of EBNA 2 to pTEFb (data not shown). To investigate the mechanism of recruitment of pTEFb to Cp further, we examined the association of the pTEFb binding protein, Brd4, with the promoter. Brd4 is recruited via binding of its bromodomains to acetylated lysines in Histones H3 and H4 [45]. We detected large increases in Histone H3 and H4 lysine acetylation at Cp in Mutu III cells and recruitment of the Histone acetyl transferase p300, previously shown to interact with EBNA 2 [9] (Figure 7). Accordingly, Brd4 was recruited to Cp in Mutu III cells over a region spanning the peaks of histone acetylation (Figure 7). We next examined whether high-level Brd4 recruitment was Cp-specific. Our data demonstrated that Brd4 was also recruited to the LMP1 and LMP2A gene promoters consistent with the peaks of Histone H3 and H4 lysine acetylation and recruitment of p300 (Figure S9). Brd4 recruitment per se could therefore not account for Cp-specific pTEFb recruitment (Figures 2 and 4). Interestingly however, experiments carried out in the presence of cellular stress revealed that pTEFb recruitment to Cp correlates with Brd4 binding. In the presence of cellular stress, such as that induced by exposure to Actinomycin D, DRB or UV, pTEFb is released from an inactive pool, where it is complexed with 7SK snRNA and the HEXIM1 protein, as part of a stress response aimed at increasing transcription factor availability. Released pTEFb then associates with Brd4 and the levels of the pTEFb/Brd4 complex are increased [34], [46]–[47]. DRB treatment has been previously shown to result in a 2-fold increase in the level of pTEFb/Brd4 complexes [34]. Consistent with these observations, we found that treatment of Mutu III cells with DRB resulted in a two-fold increase in both Brd4 and pTEFb recruitment to Cp indicating that Brd4 is responsible for recruiting pTEFb to Cp. Histone H4 acetylation was increased by DRB treatment at Cp, perhaps as a result of the protection from deacetylation provided by the preferential binding of Brd4 to acetylated Histone H4 residues. Previous studies have described inducible Brd4 recruitment via acetylated histone H4 but not acetylated histone H3 residues [48] and our data indicate that the pattern of Brd4 binding more closely resembles the profile of histone H4 rather than histone H3 acetylation (Figures 7 and S9). In sharp contrast, DRB treatment led to loss of Brd4 from the LMP1 promoter and decreases in Histone H3 and H4 acetylation (Figure 8G-J) (pTEFb is not detectably recruited to LMP1; Figure 4 and S5). Since the key difference between the Cp and LMP1 promoters is the presence of high levels of stalled pol II at Cp, these results suggest that pTEFb is efficiently recruited to Cp via Brd4 as a result of stable interactions between the pTEFb/Brd4 complex and the large numbers of stalled polymerases present at the promoter. Thus at LMP1p, in the absence of an accumulation of pol II molecules, pTEFb complexes brought in by Brd4 have little polymerase with which to stably associate and Brd4/pTEFb complex binding is not stabilized.

Interestingly, the essential EBV replication and transcription factor EBNA 1 has been shown to recruit Brd4 to a region of the latent origin of replication (OriP), that functions as an EBNA 1-dependent Cp enhancer [49]–[50]. We therefore investigated the possibility that EBNA 1 may recruit pTEFb to OriP via Brd4 and contribute to the level of pTEFb at Cp through DNA looping effects. Since EBNA 1 is expressed in Mutu I and Mutu III cells, Brd4 would be expected to be recruited to OriP by EBNA 1 in both cell types. ChIP analysis in Mutu I and Mutu III cells using primers sets close to the family of repeats (FR) element in Ori P where Brd4 was previously detected [49] revealed some Brd4 binding to Ori P in Mutu I and Mutu III cells, equivalent to that detected in the GAPDH gene (Figure S10). The level of Brd4 detected was however much lower than that present at Cp and did not appear to result in significant recruitment of pTEFb to this region of the genome (Figure S10). Our data therefore indicate that it is unlikely that pTEFb recruitment via Brd4 at OriP contributes to the level of pTEFb at Cp.

**Figure 9:**
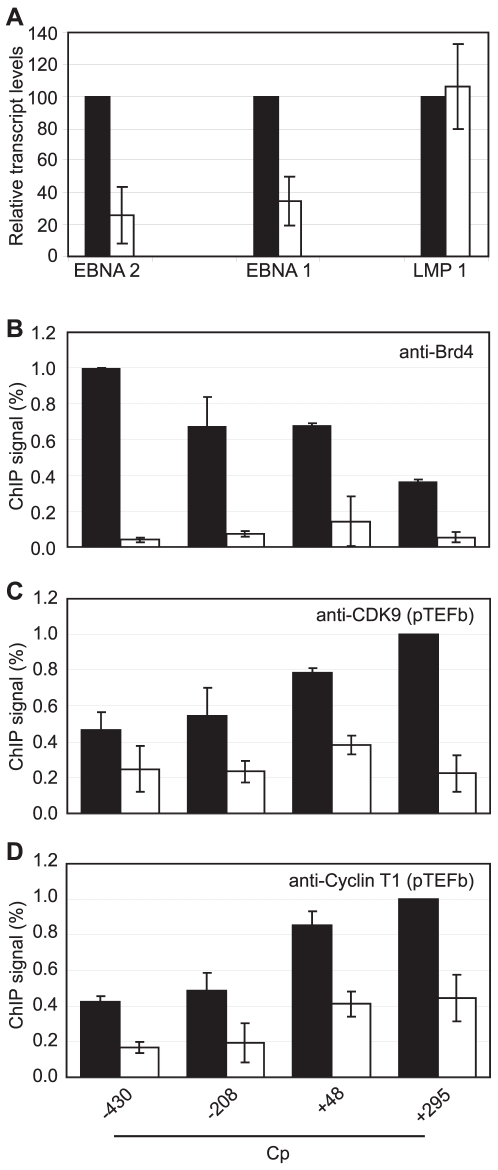
Brd4 is required for Cp transcription. (A) Transcription of the Cp-initiated transcripts EBNA 2 and EBNA 1, but not the LMP1 transcript is inhibited when Brd4 binding to chromatin is blocked in the presence of the Brd4 inhibitor JQ1. Mutu III cells were treated with 50 nM JQ1 or DMSO (control) for 48 hrs and transcript levels determined using specific Q-PCR primers and actin as an endogenous control. Normalised cDNA levels are expressed relative to 48 hr control samples. ChIP using anti-Brd4 (B), anti-CDK9 (C) and anti-cyclin T1 antibodies (D) in Mutu III cells in the absence (black bars) or presence of 50 nM JQ1 for 48 hrs (open bars). Results show the mean +/− standard deviation of two independent experiments.

We next sought to obtain direct evidence that Brd4 binding is required for Cp but not LMP1 transcription by treating Mutu III cells with the novel small molecule Brd4 bromodomain inhibitor, JQ1, previously shown to block Brd4 association with acetylated histones [51]. Strikingly, treatment with 50 nM JQ1 for 48 hrs reduced levels of Cp-initiated EBNA 2 and EBNA 1 transcripts by 74% and 65% respectively, but had no effect on LMP1 transcript levels (Figure 9A). ChIP analysis confirmed that JQ1 dramatically inhibited Brd4 association with Cp promoter regions (Figure 9). The loss of Brd4 resulted in a significant decrease in pTEFb association with Cp, consistent with Brd4-dependent recruitment of pTEFb to Cp (Figure 9). In summary, our data indicate that the binding of Brd4 to Cp is required for Cp transcription since it facilitates the stable association of pTEFb with the stalled polymerases present at Cp.

### Inhibition of pTEFb Selectively Inhibits Cp Transcription

**Figure 10:**
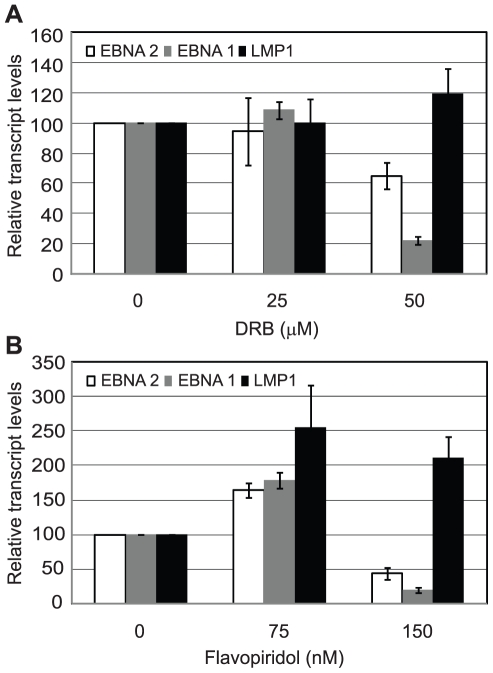
pTEFb inhibitors selectively reduce Cp transcription. Mutu III cells were treated with the indicated concentrations of DRB (A) or Flavopiridol (B) for 24 hrs and transcript levels determined using the specific Q-PCR primers indicated and actin as an endogenous control. Normalised cDNA levels are expressed relative to 24 hr control samples. Results show the mean +/− standard deviation of two independent experiments.

Since inhibition of Brd4 binding was sufficient to selectively inhibit Cp transcription presumably through reduced pTEFb recruitment, we investigated the effects of pTEFb inhibitors on Cp and LMP transcription in Mutu III cells. We have previously demonstrated that EBNA 2 activation of both Cp and LMP1 reporter constructs was inhibited by treatment with DRB or overexpression of a dominant negative form of the pTEFb kinase, CDK9 [36]. However, our current study indicates that LMP promoters *in vivo* show little detectable pTEFb recruitment (Figure 4). Consistent with the selective high-level recruitment of pTEFb to Cp *in vivo*, we found that the pTEFb inhibitors DRB and Flavopiridol were both able to inhibit Cp transcription at concentrations at which LMP1 transcription was unaffected (Figure 10). The discrepancy between our previous results and these observations is likely explained by the fact that the promoter context in transiently transfected reporter constructs differs significantly from the appropriately assembled chromatin structures found at promoters actively engaged in transcript production in latently infected cells. Our data indicate that a reduced propensity for nucleosome assembly around Cp allows high level recruitment of TFIID and establishes polymerase pausing at the constitutively active C promoter in infected cells. These Cp-specific features may not have been established in transient assays. Thus pTEFb may be important for EBNA 2-dependent Cp and LMP promoter activity in reporter assay systems, but differences in pTEFb requirements are evident in the context of latently infected cells.

## Discussion

EBV relies on the transcription of a long polycistronic mRNA to encode the nuclear antigens (EBNAs) essential for immortalization. Following initial production of EBNA-LP and EBNA-2 from a cellular factor-driven promoter (Wp) after primary infection [52]–[54], EBNA 2 activates an upstream promoter (Cp) leading to long-range transcription and the full panel of EBNA expression [2]. The molecular mechanisms behind this strategy have not been fully elucidated. Our data show that the necessity for this promoter switch goes beyond the simple advantage of utilizing a virally-controlled promoter, and may reflect a requirement to promote efficient transcriptional elongation ensuring production of the long (approximately 120 kb) primary message. Our results indicate that the specific recruitment of high levels of pTEFb to Cp in the presence of EBNA 2 is required to promote distal transcriptional elongation through serine 2 CTD phosphorylation and to overcome promoter-proximal pol II stalling induced by the high levels of the NELF/DSIF complex present at Cp. Our data suggest that Cp-specific pol II stalling may play dual positive roles in promoting transcription (i) by triggering the recruitment of pTEFb and promoting distal elongation and (ii) by maintaining a nucleosome-free region at the promoter that promotes initiation.

Our results document the presence of stalled RNA polymerase at an actively transcribing viral gene locus, unlike the situation observed at heat-shock genes, where genes temporarily in the ‘OFF’ state maintain promoter-proximally paused pol II to enable a rapid transcriptional ‘ON’ response to heat-shock that results in a re-distribution of polymerase along the gene [55]. Paused polymerase does not remain detectable when Cp is ‘OFF’ in the Mutu I cells used in this study because Cp is silenced in EBV positive Latency I cells through CpG DNA methylation, thus inhibiting transcription factor binding and pol II recruitment [56]–[57]. Recent fine-mapping confirmed Cp methylation in Mutu I cells and demonstrated a peak of 5-methyl cytosine close to Cp that increased 7-fold in Mutu I cells compared to an LCL generated from Mutu virus, where Cp is active [58].

Significantly, we demonstrate that regulation of Cp is distinct from the regulation of the latent membrane protein promoters, where only low levels of pol II, pTEFb, NELF and DSIF are detectable and serine 2 CTD phosphorylation does not substantially increase at distal regions. The EBNA 2-dependent LMP1, LMP2A and 2B genes encode transcripts of 2.8 kb, 11.7 and 8.4 kb in length (in the B95-8 EBV genome sequence NC_007605.1) so these shorter transcription units may therefore be less dependent on elongation factor function. It is worth noting that the LMP 2 transcription units can increase in size due the presence of varying numbers of ∼500 bp terminal repeat (TR) elements that are present within these genes. On entry into host cells, the EBV genome is initially in its linear form and the TR region is the site of recombination-directed genome circularization. Although the B95-8 genome sequence we have used for transcript annotation contains 4 TRs spanning 2.1 kb, TR regions of up to ∼12 kb have been reported, indicating that LMP2A transcripts can be up to 25 kb in length [59]. Nonetheless, the ∼120 kb Cp transcription unit requires pol II to elongate over considerably longer distances and our results indicate that it possesses distinct regulatory features that promote long-range transcription.

The specificity of pol II stalling at Cp appears to be driven by the presence of DNA sequences upstream of Cp that are less favourable for nucleosome assembly. These sequences encompass the TATA box and therefore allow increased access to TBP resulting in high-level recruitment of pol II in association with the pausing factors NELF and DSIF. Once pol II stalling is established at Cp, a more extensive region around the promoter is then maintained in a nucleosome-depleted state. We have previously demonstrated that pTEFb activity is required for the activation of both Cp and LMP1 promoter-reporter constructs by EBNA 2 [36]. In the present study, the pTEFb components cyclin T1 and CDK9 were virtually undetectable at either the LMP1/2B or LMP2A promoters. In the context of latently infected EBV immortalized cells we now show that endogenous Cp transcription can be selectively inhibited by the pTEFb inhibitors DRB and Flavopiridol at concentrations that do not affect LMP1 transcript production. It is likely that chromatin structure in our previous transient reporter constructs differs significantly from the chromatin context present in proliferating infected cells *in vivo* and thus pausing factor and elongation factor requirements at Cp may not have been faithfully recapitulated. The results presented in this manuscript show that nucleosome occupancy is likely to be the critical determinant that sets up Cp-specific polymerase pausing and pTEFb recruitment on endogenous EBV templates. It would be interesting to test whether Cp-specific regulatory features can be conferred by Cp promoter sequences alone by generating recombinant viruses in which LMP promoter regions are replaced with Cp promoter regions.

Since the studies described here have exclusively examined the nature of constitutive Cp transcription in established cell-lines, it will be interesting to investigate the kinetics of the establishment of polymerase stalling during primary B-cell immortalization, when transcription switches from Wp to Cp approximately 6 days post-infection [60], and the effects promoter switching has on CTD phosphorylation and the elongation properties of pol II. Interestingly, when we extended our nucleosome prediction analysis to include Wp, we found that the region around the Wp TATA box has a high probability of being occupied by nucleosomes, similar to the results obtained for the LMP promoters (Figure S11). Thus, based on our findings, Wp would be less likely to accumulate stalled polymerases as a result of increased TFIID access and pol II recruitment. The switch from Wp to Cp usage may therefore be advantageous for the virus, enabling high level pTEFb recruitment and increased efficiency of elongation. It is interesting however, that Cp-deleted viruses capable of transforming B-cells have been described and more recently rare Burkitt's lymphoma cells were identified that exclusively use Wp to drive EBNA transcription [61]–[62]. These observations suggest that Wp may be able to achieve sufficient long-range transcription required for EBNA production during immortalization *in vitro* or in certain cell backgrounds. Importantly, the W promoter is present in multiple copies in the EBV genome (e.g. 7.6 copies in the prototype Type 1 EBV strain, B95-8) and transcription initiation from a number of W promoters may be required to generate sufficient levels of downstream transcripts. The fact that Cp deletion is a rare event however, supports the notion that Cp plays a crucial role during EBV immortalization and infected cell growth *in vivo*.

Since we detected the presence of Spt5 at distal regions, it is also possible that the recruitment of DSIF may play a positive role in Cp transcription, as documented for HIV transcription. pTEFb-mediated phosphorylation of the Spt5 subunit of DSIF at promoter-proximal regions converts DSIF into a positive-acting elongation factor that travels with polymerase to promote processivity and inhibit further pausing [25], [27]. Spt5 has been shown to promote transcriptional activation by Gal4-VP16 and is recruited to the HIV-1 LTR to co-operate in the stimulation of transcriptional elongation by HIV-1 Tat [25], [63]. Further experiments involving RNA interference would be useful in dissecting the roles of DSIF in the regulation of Cp transcription.

EBV strains can be classified into two virus types (1 and 2, formerly A and B) largely based on sequence differences between the EBNA 2 genes, which share only 55% homology. Despite the prevalence of type 2 viruses in Africa and their association with BL, type 2 viruses transform resting B-cells much less efficiently than type 1 strains and differences between the EBNA 2 genes appear to be the major determinant of this property [64]. Recent work has identified cellular genes that are differentially regulated by type 1 and type 2 EBNA 2 indicating that reduced gene activation by type 2 EBNA 2 may contribute to the reduced transforming potential of type 2 viruses [65]. Since type 2 EBNA 2 also initially activates the LMP1 promoter to a reduced extent [65], it is conceivable that differences in Cp control by type 1 and type 2 EBNA 2 may also be evident. However, the results described here indicate that high-level pTEFb recruitment to Cp is driven by polymerase stalling initiated by DNA sequences that promote reduced nucleosome occupancy around Cp, increasing access to TFIID, rather than through specific properties of EBNA 2 (that may vary between strains). To investigate whether type 2 Cp sequences also possessed this property, we performed nucleosome occupancy predictions using the sequence of the type 2 viral strain AG876. We found that nucleosome occupancy at the type 2 C, LMP and W promoters was predicted to be virtually indistinguishable (data not shown) from that of the type I B95-8 strain previously examined (Figure 6A and S11). This is perhaps not surprising given the sequence similarity between the two strains throughout most of the genome. Thus type 2 Cp sequences show the same reduced likelihood of nucleosome occupancy as type 1 viruses, compared to the respective LMP and W promoter sequences, and would be just as likely to accumulate stalled polymerases and recruit high-levels of pTEFb. It is therefore unlikely that pTEFb recruitment would contribute to the reduced transforming potential of type 2 viruses, but further work is necessary to address this point unequivocally.

Since we have been unable to detect an interaction between EBNA 2 and pTEFb to date, we investigated alternative mechanisms for the recruitment of pTEFb to Cp. The double bromodomain protein, Brd4, has been shown to bind to the active form of pTEFb and recruit it to promoters to stimulate elongation [34]–[35]. Our data indicate that pol II stalling facilitates the association of Brd4-recruited pTEFb with the C promoter by providing high levels of pol II with which pTEFb can associate. In support of this hypothesis the interaction of pTEFb with Brd4 has been shown to be weak in nature and is disrupted by low salt concentrations [34]. Thus despite similar levels of Brd4 binding to Cp and the LMP gene loci, the lack of stalled pol II at LMP does not facilitate stable pTEFb binding to the transcription complex. Brd4 has been shown to play important roles in regulating viral transcription and in tethering viral genomes to chromatin [66]. Brd4 enhances HIV-1 transcription and promotes transcriptional activation of G1/S cyclin genes by murine gammaherpesvirus 68 (MHV-68) through direct interaction with MHV-68 orf73 [35], [67]. Brd4 also plays an important role in the repression of human papillomavirus transcription by the viral E2 protein and tethers bovine and human papillomavirus genomes to mitotic chromosomes [68]–[69]. Brd4 was also recently shown to bind to the EBV latent antigen EBNA-1 and to play a role in EBNA-1 activation of transcription; knock-down or overexpression of Brd4 inhibited EBNA-1 activated transcription in reporter assays [49]. It therefore appears that Brd4 may play multiple roles in the EBV life-cycle. Our data demonstrating specific inhibition of Cp-driven EBV transcription by the novel Brd4 inhibitor JQ1 highlights the potential for drug-like derivatives of this compound as anti-EBV agents. In addition, our further evidence for the role of pTEFb in promoting EBV transcription and the inhibition of Cp transcription by pTEFb inhibitors adds weight to the possible use of the pTEFb targeting anti-cancer drug, Flavopiridol, in the treatment of EBV-associated tumours.

In summary, we demonstrate that polymerase stalling may play a role in facilitating immortalization by the tumour virus EBV. High-level recruitment of pol II and associated pausing factors to the viral C promoter maintains nucleosome depletion and necessitates pTEFb recruitment to overcome pausing. This provides high levels of pTEFb to promote the distal serine 2 CTD phosphorylation required for production of the long viral transcript encoding key EBV immortalizing genes.

## Materials and Methods

### Cell Culture

ER/EB 2.5 cells [38] were maintained as described previously [36]. Mutu I (clone 179), Mutu III (clone 48), IB4 (provided by Martin Rowe), PER 142 B95-8 LCL and PER 253 B95-8 LCL (provided by Heather Long) were cultured as described [70]. For Brd4/pTEFb inhibition experiments, Mutu cells were resuspended at 5×10^5^ cells/ml and incubated in the presence of DMSO, JQ1/SGCBD01 [51] (kindly provided by Stefan Knapp, Structural Genomics Consortium, University of Oxford), DRB (Sigma) or Flavopiridol (Sigma) for 24 or 48 hours.

### ChIP Assays

ER/EB 2.5 cells were washed and resuspended at 5×10^5^ cells/ml in medium without β-estradiol. After 3 days 1 µM β-estradiol (Sigma) was added for 5 hours and chromatin prepared as described previously [36]. ER/EB 2.5 cells were treated with 100 µM DRB (or DMSO as a control) for 2 hrs as required prior to addition of β-estradiol.

Mutu cells were diluted to 5×10^5^ cells/ml 24 hrs prior to chromatin preparation and resuspended at 1×10^7^ cells/mL in fresh media before crosslinking. Cells were treated with 500 µM DRB for 2hrs prior to chromatin preparation.

ChIP methods were optimised for each target using a number of alternative strategies.

For ER/EB 2.5 cells ChIP assays were carried out as described previously [36] by overnight incubation at 4°C with 6 µg of polyclonal antibodies (anti-Pol II; N-20, anti-Spt5; H-300, Santa Cruz Biotechnology, Inc) followed by precipitation with protein A sepharose beads pre-blocked with salmon sperm DNA. EBNA 2 immunoprecipitations were carried out using 8 µg of monoclonal antibody (PE2) and an additional incubation with secondary antibodies [36]. DNA was purified using the QIAquick Gel extraction Kit (Qiagen) and eluted in 110 µl sterile millipore water. Phospho serine 2 immunoprecipitations in ER/EB 2.5 cells were carried out using a double-round ChIP protocol immunoprecipitating first pol II and then the phospho-specific form. Immune complexes from pol II precipitations were eluted and diluted by addition of 850 µl IP dilution buffer. Second round immunoprecipitations were carried out using protein sepharose A/G beads (1∶1 mix of protein A and G sepharose) preabsorbed first with rabbit anti-mouse IgM immunoglobulins (20 µg) in 500 µl IP dilution buffer overnight, and then with 25 µg anti-phospho ser 2 (H5) for 3–5 hours at 4°C. Prior to collection of immune complexes, 100 µl of a 50% slurry of antibody pre-coated beads were blocked using 350 µg salmon sperm DNA for 1 hr at 4°C. Immune complexes were collected by rotation at 4°C overnight.

ChIP assays for EBNA 2 using Mutu cell chromatin were carried out as for ER/EB 2.5 cells using 8 µg (PE2) antibodies. Pol II, Spt5, acetylated Histone H3 and acetylated Histone H4 immunoprecipitations were carried out as described previously [36] by overnight incubation of chromatin lysates with 5 µg of anti-pol II, anti-Spt5 (H-300), anti-CDK9 (H-169), anti-Cyclin T1 (H-245), anti-Brd4 (H-250) (Santa Cruz Biotechnology, Inc), anti-acetyl-Histone H3 or H4 (Millipore) antibodies. ChIP assays for core Histone H3 were carried out using 2 µg anti-Histone H3 antibody (Abcam, ab1791). For NELF-A, immunoprecipitations were carried out using a polyclonal antibody (Santa Cruz Biotechnology, Inc) and precoating protein A/G sepharose beads with 5 µg (anti-NELF-A; A20) antibody overnight. Immune complexes were collected overnight following blocking of pre-coated beads with salmon sperm DNA as above. Phospho serine 2 and 5 immunoprecipitations using Mutu cell chromatin were carried out in a single round ChIP by precoating protein A/G sepharose beads with 10 µg rabbit anti-mouse IgM overnight, prior to the addition of 25 µg H5 or 5 µg H14 antibodies and then salmon sperm DNA as above. All controls were treated identically but without addition of antibodies.

### cDNA Preparation

Cells were diluted to 5×10^5^/ml, harvested after 24 hrs and total RNA extracted using TriReagent (Sigma). RNA samples were purified using the RNeasy kit (Qiagen) and cDNA was then synthesised using the ImProm II reverse transcription system using random oligonucleotides (Promega). For Brd 4 and pTEFb inhibitor experiments, cDNA was prepared from 10^5^ cells using Power SYBR Green Cells-to-CT Kit (Applied Biosystems) according to the manufacturer's instructions.

### PCR

Quantitative PCR (Q-PCR) was performed as described previously [36] using an Applied Biosystems 7500 real-time PCR machine (95°C for 10 mins, 40 cycles at 95°C for 15 sec and 60°C for 1 min and dissociation curve analysis). For ChIP analysis, an input control standard curve was generated for each primer set (Table S1). Generally, cDNA samples were analysed using the absolute quantitation method with standard curves generated from Mutu I or Mutu III cDNA. Transcript levels were determined using Qp or Cp-specific primers [71], cDNA-specific EBNA 2, EBNA 1 (YUK) or LMP1-specific primers [72] and either the 18S rRNA Quantitect primer assay (Qiagen) or actin primers as normalization controls (Table S1). For Brd 4 inhibition experiments, Q-PCR was carried out using Power SYBR Green Cells-to-CT Kit (Applied Biosystems) and cDNA-specific EBNA 2, EBNA 1 (YUK) or LMP1-specific primers [72] with actin as the endogenous control and analysed by Relative Quantification (ddCt).

### Immunoblotting

SDS-PAGE analysis and immunoblotting was carried out as described previously [36], [70]. Blots were probed with human M.S. serum at 1/200 to detect EBNA 1 (gift from Martin Rowe), PE2 at 1/300 to detect EBNA 2 and anti-actin at 1/5000 (A-2066, Sigma). HRP-conjugated anti-mouse (Dako) or anti-rabbit antibodies (Cell Signalling Technology) were used to detect EBNA 2 and actin respectively, and HRP-conjugated protein A (1/1000, Amersham Biosciences) was used to detect EBNA 1 primary antibodies.

### Accession Numbers

The type 1 EBV genome used for primer design, transcription start sites and nucleosome predictions is the annotated sequence from the B95-8 strain (NC_007605.1). The type 2 EBV genome used was from the AG876 strain (NC_009334.1).

## Supporting Information

Figure S1 **Increased serine 2 phosphorylation on the pol II CTD is dependent on EBNA 2 activity.** (A) ChIP using anti-EBNA 2 antibodies in ER/EB 2.5 cells cultured in the absence (open bars) or presence of β-estradiol (black bars) shows functional EBNA 2 binding only in the presence of β-estradiol (B) ChIP using anti-phospho serine 2 pol II CTD antibodies detects high-level serine 2 phosphorylation only in the presence of β-estradiol. Results show the mean +/− standard deviation of a minimum of three independent experiments carried out using at least 2 chromatin batches.
(PDF)

Figure S2 **The Q promoter drives EBNA-1-only expression in Mutu I cells.** (A) ChIP using anti-acetylated histone H3 antibodies and Mutu I (open bars) and Mutu III cell chromatin (black bars). Results show the mean +/− standard deviation of a minimum of three independent experiments carried out using at least 2 chromatin batches. Numbers indicate the 5′ end of the forward primer relative to the Cp transcription start site in the annotated EBV sequence (NC_007605.1). Qp is located at +38800 relative to the Cp transcription start site. (B) PCR amplification of Qp-specific and Cp-specific transcripts. Akata cells (Qp only) and the PER 253 LCL (Cp only) served as positive controls for Qp and Cp usage respectively. The IB4 LCL has a deletion upstream of Cp so is negative for Qp and Cp transcripts. Qp or Cp signals were normalised to 18S rRNA primer signals. (C) Western blot analysis of whole cell lysates of Mutu I and Mutu III cells. Blots were probed with M.S. human serum to detect EBNA 1, PE2 to detect EBNA 2 and re-probed with anti-actin antibodies as a loading control.
(PDF)

Figure S3 **DRB treatment of Mutu III and ER/EB 2.5 cells inhibits CTD phosphorylation.** (A) ChIP using anti-phospho serine 5 pol II CTD antibodies in Mutu III cells minus (open bars) or plus 500 µM DRB (black bars). (B) ChIP using anti-TBP antibodies in Mutu III cells −/+ DRB. (C) ChIP using anti-phospho serine 2 pol II CTD antibodies in ER/EB 2.5 cells cultured in the presence of β-estradiol and in the absence (open bars) or presence (black bars) of 100 µM DRB. (D) ChIP using anti-pol II antibodies in ER/EB 2.5 cells cultured in the presence of β-estradiol and in the absence or presence of DRB.
(PDF)

Figure S4 **Pausing factor recruitment is dependent on the function of EBNA 2.** ChIP using anti-Spt5 (DSIF) antibodies in ER/EB 2.5 cells cultured in the absence (open bars) or presence of β-estradiol (black bars) detects significant DSIF recruitment only in the presence of functional EBNA 2.
(PDF)

Figure S5 **Pol** **II is not paused at the LMP 2A promoter.** Results show the mean +/− standard deviation of four independent pol II ChIP experiments using Mutu I (open bars) and Mutu III cell chromatin (black bars). Percentage input signals, after subtraction of no antibody controls, are expressed for comparison purposes relative to the highest signal obtained using Cp-specific primers.
(PDF)

Figure S6 **Low level pol II and elongation factor recruitment at the LMP gene locus.** (A) Primers across the LMP locus are as in Figure 4. ChIP results show the mean +/− standard deviation of a minimum of two independent experiments using Mutu I (open bars) and Mutu III cell chromatin (black bars). Percentage input signals, after subtraction of no antibody controls, are expressed for comparison purposes relative to the highest signal obtained using Cp-specific primers. (B) ChIP using anti-phospho serine 2 pol II CTD antibodies. (C) ChIP using anti-phospho serine 5 pol II CTD antibodies (D) ChIP using anti-cyclin T1 antibodies. (E) ChIP using anti-Spt5 antibodies.
(PDF)

Figure S7 **Low level pol II and elongation factor recruitment at LMP genes in an LCL.** ChIP carried out in an EBV immortalised LCL (PER 253 B95-8 LCL). Primers across the LMP locus are as in Figure 4. Results show the mean +/− standard deviation of a minimum of three independent experiments. Percentage input signals, after subtraction of no antibody controls, are expressed for comparison purposes relative to the highest signal obtained using Cp-specific primers. (A) ChIP using anti-pol II antibodies. (B) ChIP using anti-Spt5 antibodies. (C) ChIP using anti-NELF A antibodies.
(PDF)

Figure S8 **Cp-initiated EBNA 2 and EBNA 1 transcript levels are similar to those of LMP1.** Transcript levels from cDNA prepared at the same time from Mutu I, Mutu III, PER 253 B95.8 LCL and PER 142 B95.8 LCL were determined using specific Q-PCR primers to EBNA 2, Cp-initiated EBNA 1 (YUK spliced) and LMP1. Transcript quantities were determined using the absolute quantitation method and a cDNA standard curve and divided by actin quantities as a normalization control. Results show mean +/− standard deviation of Q-PCR duplicates from a representative experiment. Note that EBNA 1 transcripts initiate from Qp in Mutu I cells (Figure S2) and are not detected by the YUK EBNA 1 primer set used here.
(PDF)

Figure S9 **Brd4 is recruited to LMP gene promoters.** Results show the mean +/− standard deviation of a minimum of three independent experiments using Mutu I (open bars) and Mutu III cell chromatin (black bars). LMP gene locus primers are as in Figure 4. Percentage input signals, after subtraction of no antibody controls, are expressed for comparison purposes relative to the highest signal obtained using Cp-specific primers. (A) ChIP using anti-acetyl Histone H3 antibodies. (B) ChIP using anti-acetyl Histone H4 antibodies. (C) ChIP using anti-p300 antibodies. (D) ChIP using anti-Brd 4 antibodies.
(PDF)

Figure S10 **pTEFb is not recruited to OriP at high levels.** Results show the mean percentage input signal (after subtraction of the no antibody control signal) +/− standard deviation of two independent experiments using Mutu I (open bars) and Mutu III cell chromatin (black bars). (A) ChIP using anti-Brd4 antibodies. (B) ChIP using anti-CDK9 antibodies (C) ChIP using anti-cyclin T1 antibodies. Cp analysis was carried out with the primer set that gave the highest signal for each transcription factor. Ori P primers are adjacent to the EBNA 1 binding element (family of repeats, FR) (Table S1).
(PDF)

Figure S11 **Nucleosome occupancy prediction analysis at Wp resembles LMP genes.** The probability of nucleosome occupancy (P occupancy) at regions proximal to Cp, LMP1p, LMP 2Ap and Wp was predicted using tools available at http://genie.weizmann.ac.il/software/nucleo_prediction.html [44]. Cp, LMP1p, LMP 2Ap and Wp TATA boxes are located at −31 to −26, −32 to −27, −28 to −23 and −31 to −26 relative to the transcription start sites (+1).
(PDF)

Table S1 **Real-time PCR primers.** ^a^ Primer locations are given relative to the start of the relevant mRNA sequence in the annotated EBV sequence (NC_007605.1) or the GAPDH gene. ^b^ Numbers relate to the annotated EBV sequence (NC_007605.1). ^c^ Primers are located in the W repeat region which contains on average 7.6 repeats of an estimated 3072bp sequence. ^d^ Primer sequences obtained from Prof. Paul Lieberman, The Wistar Institute, Philadelphia, USA. ^e^ Primer locations for the LMP1 gene located in the reverse orientation are given in parentheses. ^f^ The LMP1 polyA is located at 166483 to 166488 so MW 361 is outside of the transcription unit. ^g^ Actin primers span exon 3 to 4. Nucleotide positions for chromosome 7 are shown (BC002409).
(PDF)
