# Supplementary figures and images for: RNA Polymerase II Stalling Promotes Nucleosome Occlusion and pTEFb Recruitment to Drive Immortalization by Epstein-Barr Virus
Source: PLoS Pathog. 2011 Oct 27;7(10):e1002334. doi: 10.1371/journal.ppat.1002334 (PMC3203192; doi:10.1371/journal.ppat.1002334)

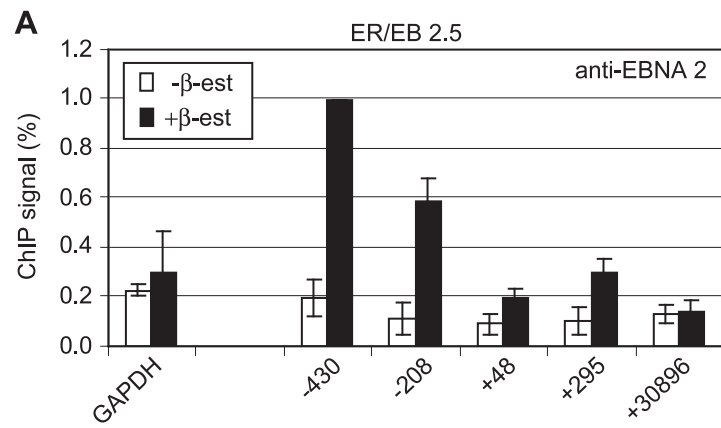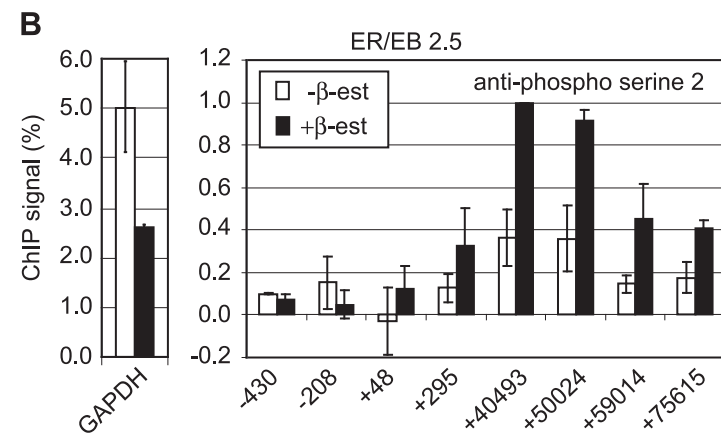

Supplement: Figure S1 — Increased serine 2 phosphorylation on the pol II CTD is dependent on EBNA 2 activity. (A) ChIP using anti-EBNA 2 antibodies in ER/EB 2.5 cells cultured in the absence (open bars) or presence of β-estradiol (black bars) shows functional EBNA 2 binding only in the presence of β-estradiol (B) ChIP using anti-phospho serine 2 pol II CTD antibodies detects high-level serine 2 phosphorylation only in the presence of β-estradiol. Results show the mean +/− standard deviation of a minimum of three independent experiments carried out using at least 2 chromatin batches. (PDF) [file ppat.1002334.s001.pdf]

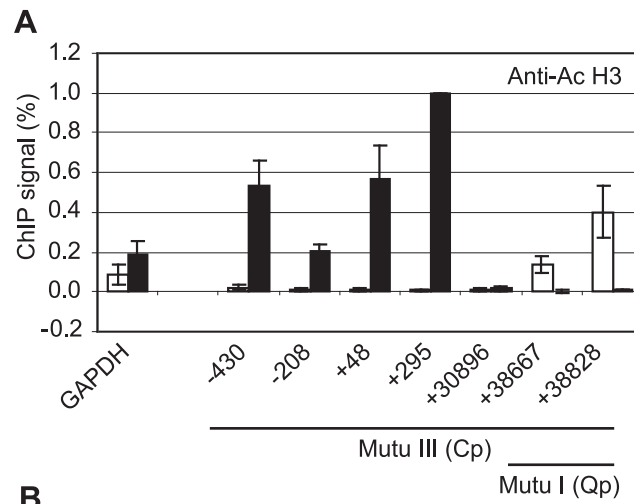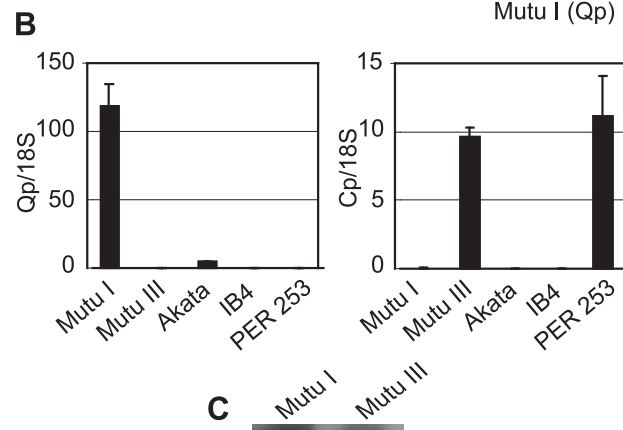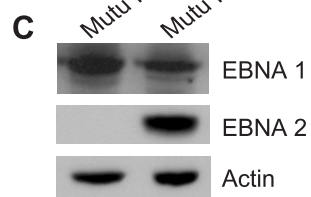

Supplement: Figure S2 — The Q promoter drives EBNA-1-only expression in Mutu I cells. (A) ChIP using anti-acetylated histone H3 antibodies and Mutu I (open bars) and Mutu III cell chromatin (black bars). Results show the mean +/− standard deviation of a minimum of three independent experiments carried out using at least 2 chromatin batches. Numbers indicate the 5′ end of the forward primer relative to the Cp transcription start site in the annotated EBV sequence (NC_007605.1). Qp is located at +38800 relative to the Cp transcription start site. (B) PCR amplification of Qp-specific and Cp-specific transcripts. Akata cells (Qp only) and the PER 253 LCL (Cp only) served as positive controls for Qp and Cp usage respectively. The IB4 LCL has a deletion upstream of Cp so is negative for Qp and Cp transcripts. Qp or Cp signals were normalised to 18S rRNA primer signals. (C) Western blot analysis of whole cell lysates of Mutu I and Mutu III cells. Blots were probed with M.S. human serum to detect EBNA 1, PE2 to detect EBNA 2 and re-probed with anti-actin antibodies as a loading control. (PDF) [file ppat.1002334.s002.pdf]

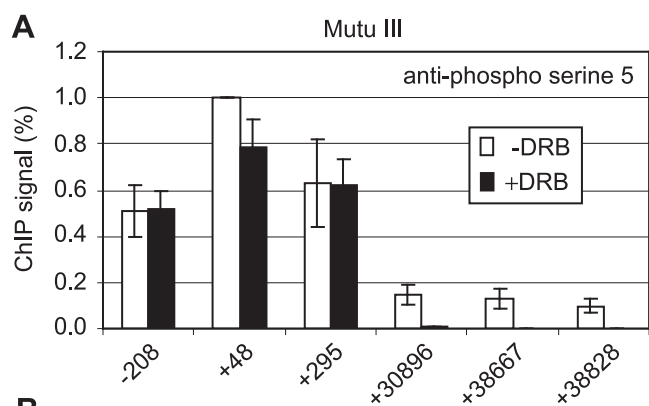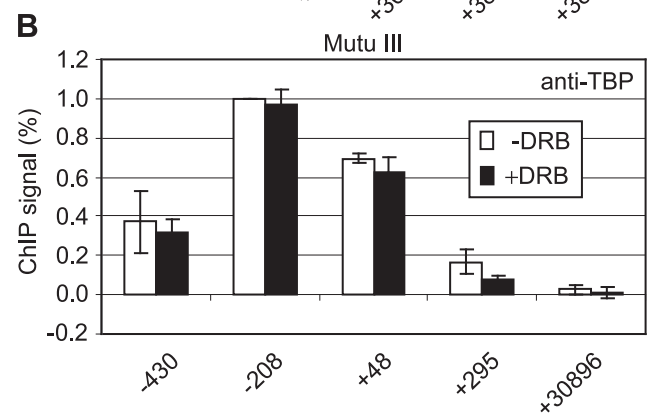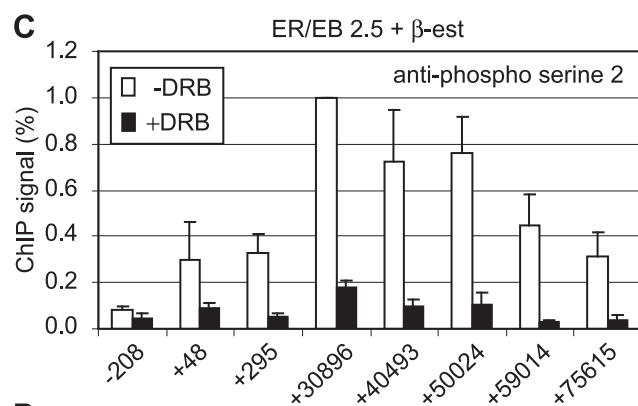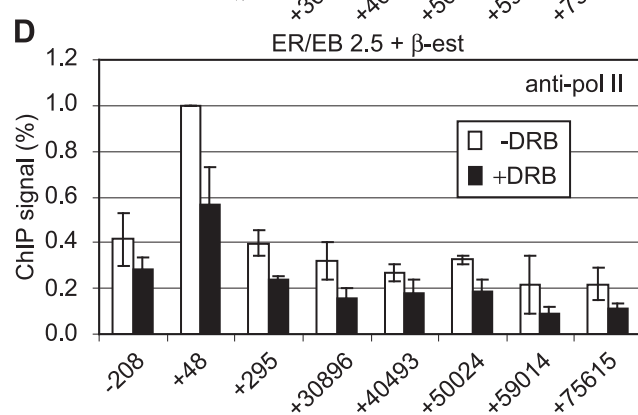

Supplement: Figure S3 — DRB treatment of Mutu III and ER/EB 2.5 cells inhibits CTD phosphorylation. (A) ChIP using anti-phospho serine 5 pol II CTD antibodies in Mutu III cells minus (open bars) or plus 500 µM DRB (black bars). (B) ChIP using anti-TBP antibodies in Mutu III cells −/+ DRB. (C) ChIP using anti-phospho serine 2 pol II CTD antibodies in ER/EB 2.5 cells cultured in the presence of β-estradiol and in the absence (open bars) or presence (black bars) of 100 µM DRB. (D) ChIP using anti-pol II antibodies in ER/EB 2.5 cells cultured in the presence of β-estradiol and in the absence or presence of DRB. (PDF) [file ppat.1002334.s003.pdf]

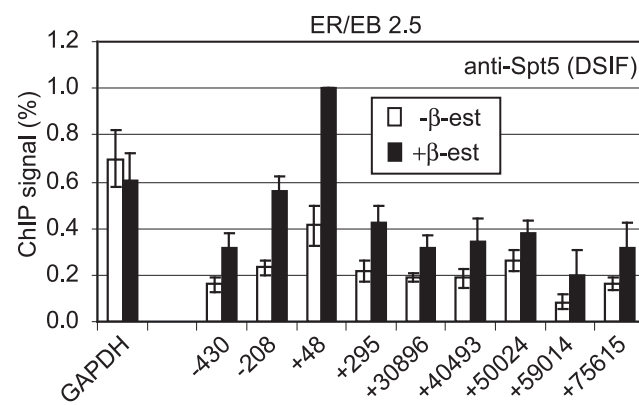

Supplement: Figure S4 — Pausing factor recruitment is dependent on the function of EBNA 2. ChIP using anti-Spt5 (DSIF) antibodies in ER/EB 2.5 cells cultured in the absence (open bars) or presence of β-estradiol (black bars) detects significant DSIF recruitment only in the presence of functional EBNA 2. (PDF) [file ppat.1002334.s004.pdf]

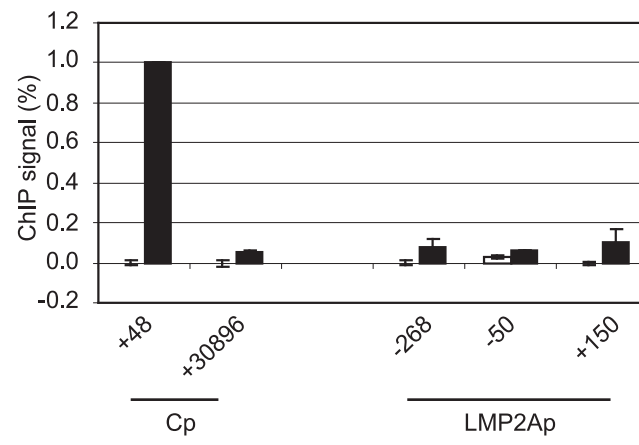

Supplement: Figure S5 — Pol II is not paused at the LMP 2A promoter. Results show the mean +/− standard deviation of four independent pol II ChIP experiments using Mutu I (open bars) and Mutu III cell chromatin (black bars). Percentage input signals, after subtraction of no antibody controls, are expressed for comparison purposes relative to the highest signal obtained using Cp-specific primers. (PDF) [file ppat.1002334.s005.pdf]

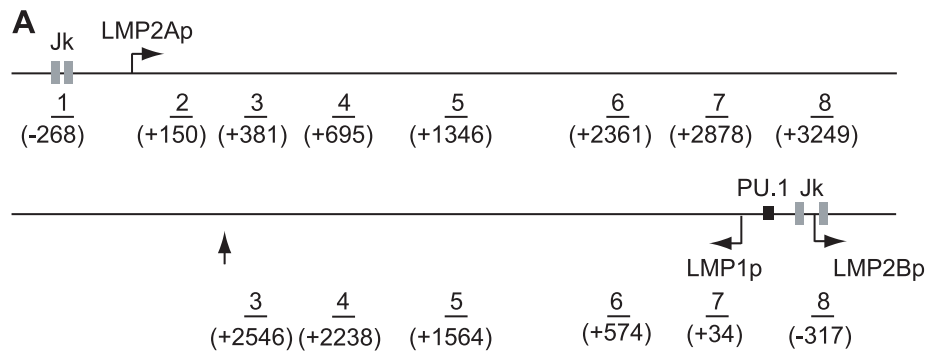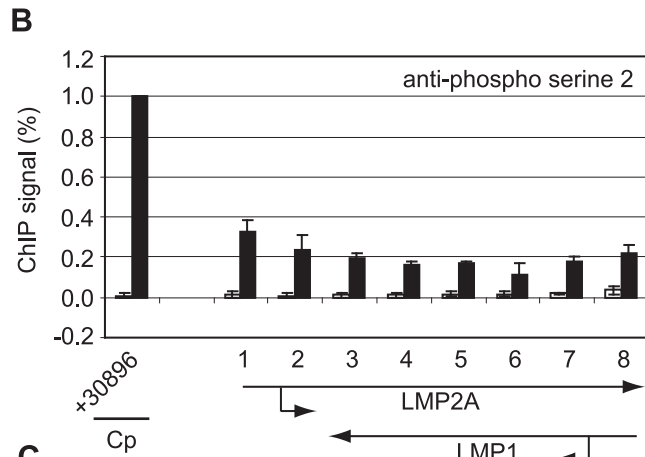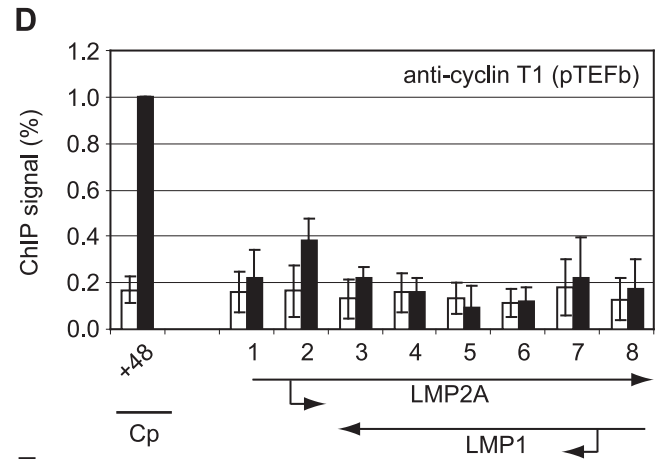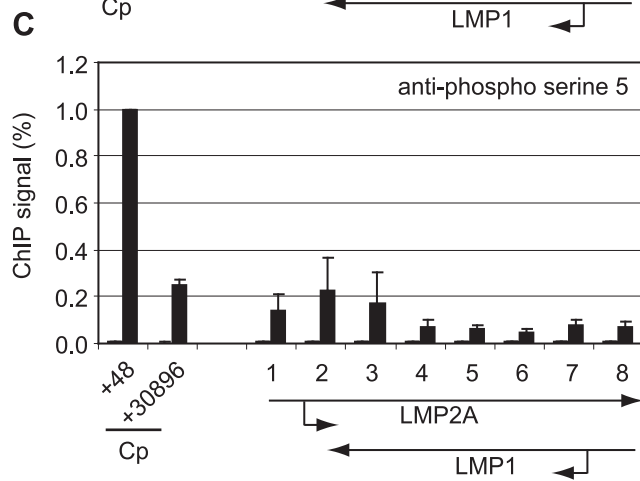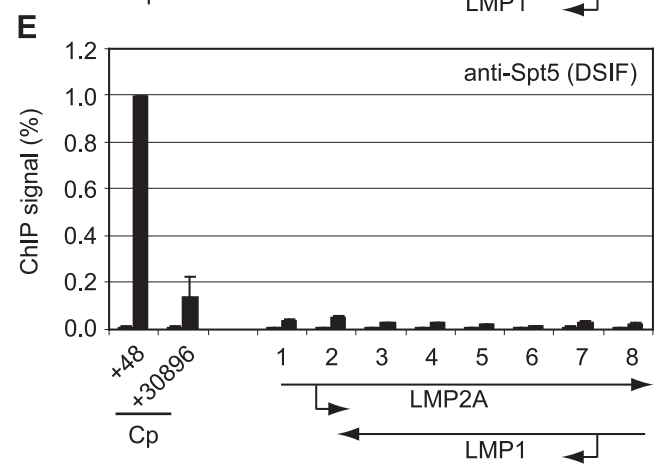

Supplement: Figure S6 — Low level pol II and elongation factor recruitment at the LMP gene locus. (A) Primers across the LMP locus are as in Figure 4. ChIP results show the mean +/− standard deviation of a minimum of two independent experiments using Mutu I (open bars) and Mutu III cell chromatin (black bars). Percentage input signals, after subtraction of no antibody controls, are expressed for comparison purposes relative to the highest signal obtained using Cp-specific primers. (B) ChIP using anti-phospho serine 2 pol II CTD antibodies. (C) ChIP using anti-phospho serine 5 pol II CTD antibodies (D) ChIP using anti-cyclin T1 antibodies. (E) ChIP using anti-Spt5 antibodies. (PDF) [file ppat.1002334.s006.pdf]

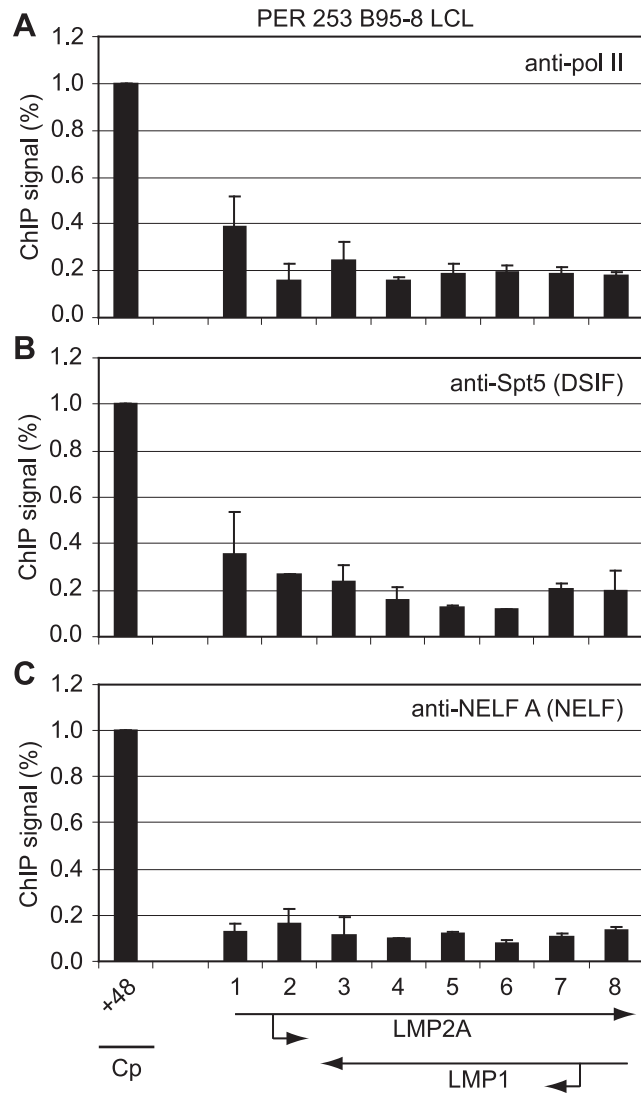

Supplement: Figure S7 — Low level pol II and elongation factor recruitment at LMP genes in an LCL. ChIP carried out in an EBV immortalised LCL (PER 253 B95-8 LCL). Primers across the LMP locus are as in Figure 4. Results show the mean +/− standard deviation of a minimum of three independent experiments. Percentage input signals, after subtraction of no antibody controls, are expressed for comparison purposes relative to the highest signal obtained using Cp-specific primers. (A) ChIP using anti-pol II antibodies. (B) ChIP using anti-Spt5 antibodies. (C) ChIP using anti-NELF A antibodies. (PDF) [file ppat.1002334.s007.pdf]

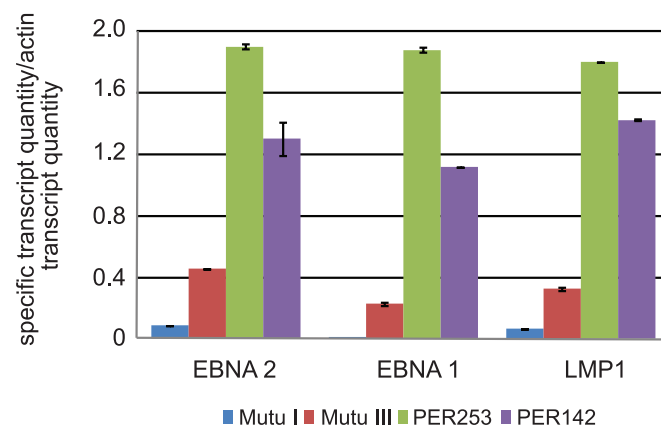

Supplement: Figure S8 — Cp-initiated EBNA 2 and EBNA 1 transcript levels are similar to those of LMP1. Transcript levels from cDNA prepared at the same time from Mutu I, Mutu III, PER 253 B95.8 LCL and PER 142 B95.8 LCL were determined using specific Q-PCR primers to EBNA 2, Cp-initiated EBNA 1 (YUK spliced) and LMP1. Transcript quantities were determined using the absolute quantitation method and a cDNA standard curve and divided by actin quantities as a normalization control. Results show mean +/− standard deviation of Q-PCR duplicates from a representative experiment. Note that EBNA 1 transcripts initiate from Qp in Mutu I cells (Figure S2) and are not detected by the YUK EBNA 1 primer set used here. (PDF) [file ppat.1002334.s008.pdf]

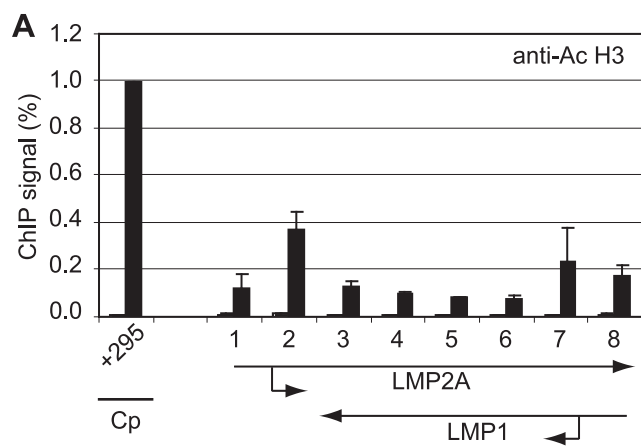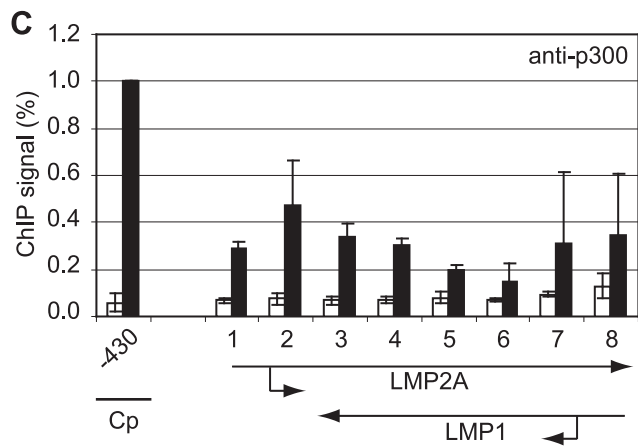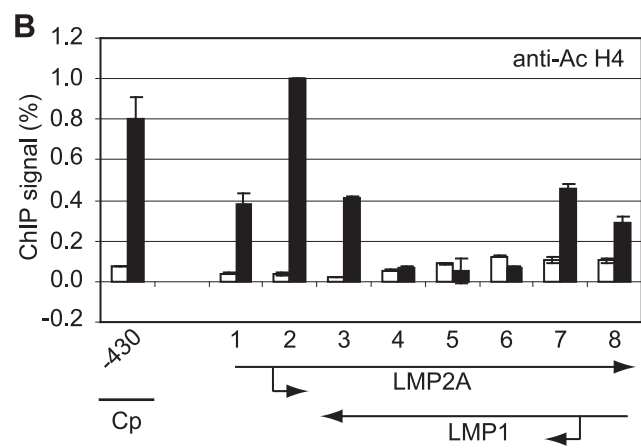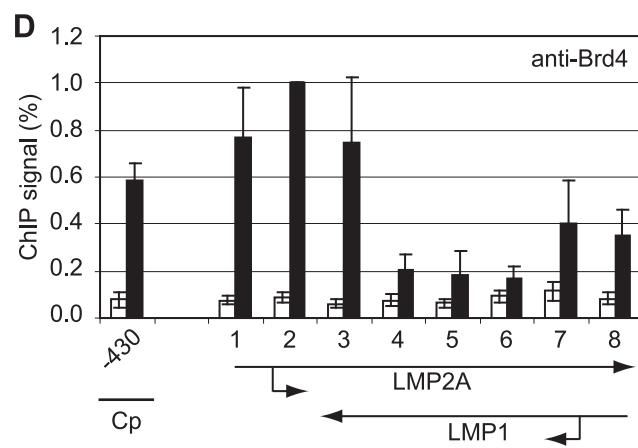

Supplement: Figure S9 — Brd4 is recruited to LMP gene promoters. Results show the mean +/− standard deviation of a minimum of three independent experiments using Mutu I (open bars) and Mutu III cell chromatin (black bars). LMP gene locus primers are as in Figure 4. Percentage input signals, after subtraction of no antibody controls, are expressed for comparison purposes relative to the highest signal obtained using Cp-specific primers. (A) ChIP using anti-acetyl Histone H3 antibodies. (B) ChIP using anti-acetyl Histone H4 antibodies. (C) ChIP using anti-p300 antibodies. (D) ChIP using anti-Brd 4 antibodies. (PDF) [file ppat.1002334.s009.pdf]

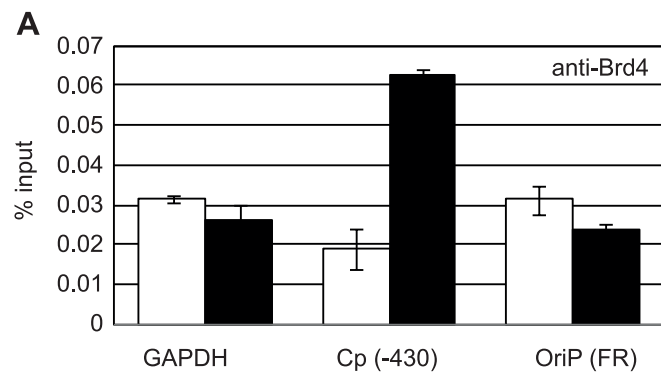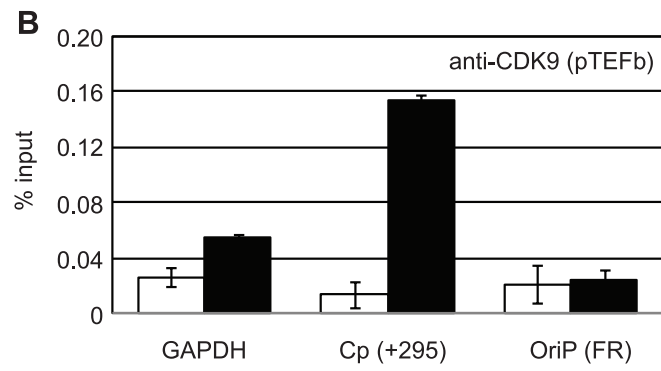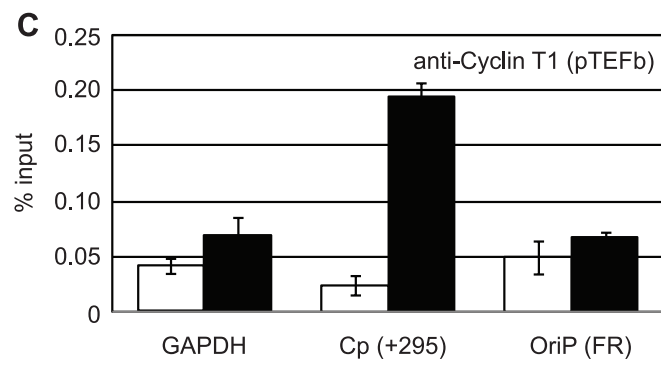

Supplement: Figure S10 — pTEFb is not recruited to OriP at high levels. Results show the mean percentage input signal (after subtraction of the no antibody control signal) +/− standard deviation of two independent experiments using Mutu I (open bars) and Mutu III cell chromatin (black bars). (A) ChIP using anti-Brd4 antibodies. (B) ChIP using anti-CDK9 antibodies (C) ChIP using anti-cyclin T1 antibodies. Cp analysis was carried out with the primer set that gave the highest signal for each transcription factor. Ori P primers are adjacent to the EBNA 1 binding element (family of repeats, FR) (Table S1). (PDF) [file ppat.1002334.s010.pdf]

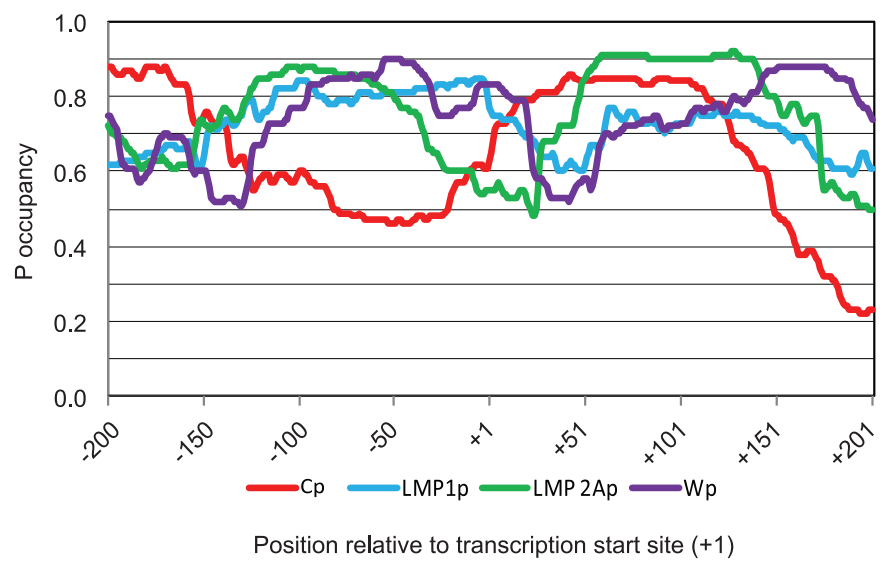

Supplement: Figure S11 — Nucleosome occupancy prediction analysis at Wp resembles LMP genes. The probability of nucleosome occupancy (P occupancy) at regions proximal to Cp, LMP1p, LMP 2Ap and Wp was predicted using tools available at http://genie.weizmann.ac.il/software/nucleo_prediction.html [44]. Cp, LMP1p, LMP 2Ap and Wp TATA boxes are located at −31 to −26, −32 to −27, −28 to −23 and −31 to −26 relative to the transcription start sites (+1). (PDF) [file ppat.1002334.s011.pdf]
