# Supplementary material for: RNA Polymerase II Stalling Promotes Nucleosome Occlusion and pTEFb Recruitment to Drive Immortalization by Epstein-Barr Virus
Source: PLoS Pathog. 2011 Oct 27;7(10):e1002334. doi: 10.1371/journal.ppat.1002334 (PMC3203192; doi:10.1371/journal.ppat.1002334)
Supplement: Table S1 — Real-time PCR primers. a Primer locations are given relative to the start of the relevant mRNA sequence in the annotated EBV sequence (NC_007605.1) or the GAPDH gene. b Numbers relate to the annotated EBV sequence (NC_007605.1). c Primers are located in the W repeat region which contains on average 7.6 repeats of an estimated 3072bp sequence. d Primer sequences obtained from Prof. Paul Lieberman, The Wistar Institute, Philadelphia, USA. e Primer locations for the LMP1 gene located in the reverse orientation are given in parentheses. f The LMP1 polyA is located at 166483 to 166488 so MW 361 is outside of the transcription unit. g Actin primers span exon 3 to 4. Nucleotide positions for chromosome 7 are shown (BC002409). (PDF) [file ppat.1002334.s012.pdf]

Name	Location[a]	B95-8 sequence number[b]	Sequence (5' to 3')
Cp primer sets			
Forward (MW 428)	-1409 to -1392	9926 to 9943	GGAGGAAGTCATGCCACA
Reverse (MW 429)	-1310 to -1293	10025 to 10042	AGGAACCTGTGGCACCTG
Forward (MW 70)	-430 to -410	10905 to 10925	CCTAGGCCAGCCAGAGATAAT
Reverse (MW 71)	-356 to -337	10979 to 10998	AGATAGCACTCGACGCACTG
Forward (MW 133)	-208 to -189	11127 to 11146	ACCTTAGAGGTGGAGCAACG
Reverse (MW 134)	-114 to -96	11221 to 11240	GGCGAATTAACTGAGCTTGC
Forward (MW 432)	-107 to -89	11228 to 11246	CAGTTAATTCGCCCACGAC
Reverse (MW 433)	-23 to -2	11312 to 11333	AATTTGCAGCAGAACACAGAGT
Forward (MW 155)	+48 to +67	11383 to 11402	CATCGCAGGGTTCTTACCAT
Reverse (MW 156)	+148 to +167	11483 to 11502	CCTCAGGAGGCCCTTAGACT
Forward (MW 157)	+295 to +313	11630 to 11649	GAAGAAACAGCCTCCTGCAC
Reverse (MW 158)	+387 to +406	11722 to 11741	TTCAGTGCCCAGATTCATGT
Forward (MW 274)	+2590 to +2605 (first repeat)[c]	13925 to 13940[c]	CCAGGCCTGCCAAAGA
Reverse (MW 275)	+2636 to +2654 (first repeat)[c]	13971 to 13989[c]	CTGTTACTGCACCCGCTTT
Forward (MW 226)	+30896 to +30916[d]	42231 to 42251	GCGTTATCATGTGTTGCTTGA
Reverse (MW 227)	+30973 to +30994[d]	42308 to 42329	AGATCCACATGTTCGGTGTG
Forward (MW 280)	+38667 to +38686	50002 to 50021	CTGTCACCACCTCCCTGATA
Reverse (MW 281)	+38712 to +38731	50047 to 50066	GAACACTCCCTCAGTGGTCA
Forward (MW 282)	+38828 to +38847	50163 to 50182	GATGGCGGGTAATACATGCT
Reverse (MW 283)	+38903 to +38922	50238 to 50257	CAAACATACACCGTGCGAAA
Forward (MW 288)	+40493 to +40512	51828 to 51847	TCGAAAGACTCGGAGTAGCC
Reverse (MW 289)	+40542 to +40557	51878 to 51892	TCCCTGGTGGAGACC
Forward (MW 290)	+50024 to +50044	61359 to 61379	GACCCATAGCAGAGACCTGAG
Reverse (MW 291)	+50071 to +50088	61407 to 61423	CGGCCGAGTATGAGCAG
Forward (MW 161)	+59014 to +59033	70349 to 70368	TGAGGCATCCGATATGACTG
Reverse (MW 162)	+59083 to +59104	70418 to 70439	CTCCACCTGTGTAACCTCACTG
Forward (MW 266)	+75615 to +75634	86950 to 86969	AACTGTGTGGCTCTCTGCAC
Reverse (MW 267)	+75696 to +75714	87031 to 87049	TTGCAGCTGGACATGACAC
LMP2A / (LMP1) primer sets[e]			
Forward (MW 357)	-268 to –246	165771 to 165793	GATAGCCTCGCGACTCGTGGGAA
Reverse (MW 358)	-205 to -185	165834 to 165854	AATCTTCACACACTGCTGCTG
Forward (MW 526)	-50 to -30	165991 to 166011	CGGTTTCAGCATCACAGGT
Reverse (MW 527)	+12 to +34	166051 to 166073	GAAATGCTCTCTGAGAAACAAGG
Forward (MW 359)	+150 to +170	166189 to 166209	CCAATATCCATCTGCTTCTGG
Reverse (MW 360)	+208 to +231	166247 to 166270	GGCTCTTCATTAGATTCACGTTC
Forward (MW 361)	+381 to +406 (+2637 to +2611)[f]	166420 to 166446	CTCATCTCAACACATATATGAAGAAGC
Reverse (MW 362)	+451 to +472 (+2567 to +2546)	166490 to 166511	TTGATGTGACTTGTGATGCAAT
Forward (MW 144)	+695 to +712 (+2323 to +2306)	166734 to 166751	GGACACGCTCCTTCTTGG
Reverse (MW 143)	+759 to +780 (+2259 to +2238)	166798 to 166819	ACTGGCTGGATTCTACGCTACT
Forward (MW 150)	+1346 to +1367 (+1672 to +1651)	167385 to 167406	GTGTTGTGCAGAGGTCTGATG
Reverse (MW 149)	+1435 to +1454 (+1583 to +1564)	167474 to 167493	CCTTCACACACCACACAGGT
Forward (MW 148)	+2361 to +2381 (+657 to +637)	168400 to 168420	TGAGCAGGATGAGGTCTAGGA
Reverse (MW 147)	+2424 to +2444 (+594 to +574)	168463 to 168483	GGAGATTCTCTGGCGACTTG
Forward (MW 140)	+2878 to +2898 (+140 to +120)	168917 to 168937	AGAGGAGGAGAAGGAGAGCAA
Reverse (MW 139)	+2966 to +2984 (+52 to +34)	169005 to 169023	CCTGAGGATGGAACACGAC
Forward (MW 311)	+3249 to +3266 (-231 to -248)	169288 to 169305	GGCCAAGTGCAACAGGAA
Reverse (MW 310)	+3315 to +3335 (-297 to -317)	169354 to 169374	GCAGATTACACTGCCGCTTC

OriP primer set			
Forward (MW 617)		7036 to 7055	GGAGGAGAAGAGAGGCTTCC
Reverse (MW 618)		7106 to 7125	AATAGCGGACAAGCCGAATA
GAPDH primer set			
Forward (MW 208)	+1948 to +1968		CTCATGCCTTCTTGCCTCTT
Reverse (MW 209)	+2027 to +2048		TTGATGGCAACAATATCCACTT
Actin cDNA primer set			
Forward (MW 418)	5568881 to 5568901 [g]		CTGGCACCACACCTTCTACA
Reverse (MW 419)	5568314 to 5568294		TTGCTATCCAGGCTGTGCTA
